# Physical Activity in Short Daily Home Dialysis versus to In-Center Hemodialysis: Results from the Self-convecTive Evaluation of Short Daily Home Dialysis on Physical Activity Study

**DOI:** 10.34067/KID.0000001246

**Published:** 2026-06-18

**Authors:** Natalia-Guillemina Target, Pierre-Antoine Michel, Cecile Courivaud, Michel Thomas, Fabien Le Floc’h, Bernard Canaud

**Affiliations:** 1 Service de Néphrologie, Pôle Santé du Confluent, Association ECHO, Rezé. 6 rue de la Gare, Rezé, France; 2 Service de Néphrologie et Dialyses, Hôpital TENON, Assistance Publique - Hôpitaux de Paris, Paris, France; 3 Service de Néphrologie, Dialyse et Transplantation, CHU Besançon, 3, Boulevard Fleming, Besançon, France; 4 Physidia, R&D, 11-13 Boulevard des Bretonnières, Saint-Barthélemy d’Anjou, France; 5 Monitoring Force Group, 38, Rue Jean Mermoz- bat.D, Maisons Laffitte Cedex, France; 6 School of Medicine, Montpellier University I, Montpellier, France

**Keywords:** CKD, chronic renal insufficiency, dialysis, nutrition, home hemodialysis

## Abstract

**Key Points:**

- Short daily home hemodialysis preserves physical activity, with ≈30% higher step counts and no dialysis-day decline compared with in-center hemodialysis.
- Short daily home hemodialysis is associated with a reduced treatment burden despite more frequent sessions.
- Short daily home hemodialysis delivers effective and more physiological dialysis, achieving better middle-molecule clearance and preserved nutritional status.

**Background:**

Short daily home dialysis (SDHD) has been proposed as a patient-centered alternative to conventional in-center hemodialysis (ICHD).

**Methods:**

The multicenter Self-convecTive Evaluation of short daily home dialysis on Physical Activity observational and prospective cohort compared SDHD with ICHD over 12 months in France and Belgium. The primary end point was average daily step count, assessed using wearable activity trackers. Secondary outcomes included sleep, sedentary time, recovery after dialysis, laboratory parameters, quality of life, and treatment burden.

**Results:**

A total of 152 patients were enrolled (82 SDHD, 70 ICHD). At baseline, SDHD patients demonstrated 28% higher activity levels (6673 versus 4768 steps/d), a difference sustained throughout follow-up. Physical activity in ICHD patients was significantly lower on dialysis days, whereas SDHD patients maintained stable patterns. Sleep duration was greater un SDHD (up to 405 minutes/d) and lower in ICHD (to 202 minutes/d). Sedentary time remained consistently lower, and recovery time tended to be shorter in SDHD (60 versus 120 minutes). Laboratory analyses showed lower predialysis *β*2-microglobulin and preserved nutritional markers in SDHD, despite similar anemia and metabolic control.

**Conclusions:**

Compared with ICHD, SDHD was associated with sustained preservation of physical activity, better postdialysis recovery, improved sleep, and reduced sedentary burden, supporting its use as a safe and patient-centered modality.

## Visual Abstract

**Figure:**
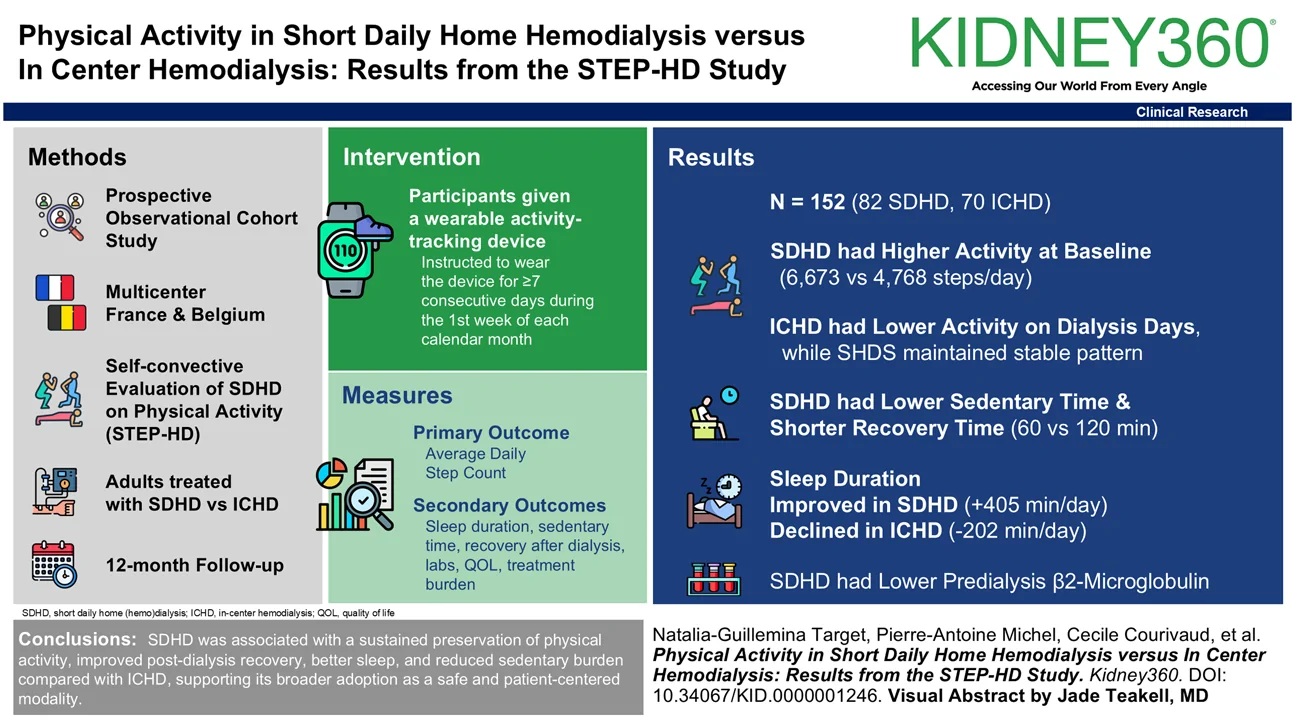

## Introduction

KRT by dialysis for patients with kidney failure is associated with a substantial and multifaceted burden.^1^ For patients, it significantly affects quality of life (QoL). From a clinical perspective, outcomes remain suboptimal, with persistently high rates of morbidity and mortality.^2^ At the societal level, dialysis requires substantial and growing resources, including dialysis machines, treatment facilities, and health care personnel, to accommodate the growing demand associated with the expanding kidney failure population.^1,3^ Economically, dialysis represents a major health care expenditure, accounting for an estimated 1.5%–2% of total health care spending globally with variation across countries.^4,5^ Furthermore, the current dialysis population is characterized by advanced age and a high prevalence of comorbidities, particularly diabetes and cardiovascular disease, necessitating more personalized treatment schedules and additional medical support.^6,7^

In this context, several studies have highlighted that dialysis modality (*e.g*., hemodiafiltration versus conventional hemodialysis), treatment schedule (intensive or more frequent versus standard thrice-weekly sessions), and treatment setting (home-based versus in-center care) may influence not only clinical outcomes but also patient-reported outcomes, including QoL and overall treatment costs.^8–13^ These findings underscore the potential value of tailoring dialysis strategies to individual patient profiles within a patient-centered care approach, thereby enhancing the effectiveness and sustainability of KRT.

The objective of this study was to evaluate the impact on physical activity of SDHD compared with in-center conventional (in-center hemodialysis [ICHD]) thrice-weekly hemodialysis.^14^ Additional end points included treatment efficacy, tolerance, acceptability, and potential side effects.

## Methods

### Study Design

Self-convecTive Evaluation of Home hemodialysis on Physical Activity study is an observational, longitudinal, prospective, international, multicentric, cohort study comparing SDHD to ICHD patients, conducted with metropolitan French and Belgian hemodialysis centers.

The decision and modalities of SDHD or ICHD were made independently of the study. Participants were prospectively enrolled between October 2018 and June 2021 and followed for up to 12 months, until kidney transplant or withdrawal of consent, whichever occurred first. The trial is registered with ClinicalTrials.gov, number NCT03737578, and the protocol was published in BioMed Central Nephrology in 2020.^14^

### Study Population

Eligible participants had to be 18 years or older, affiliated with a social security system, and must have provided written informed consent. Eligible SDHD patients were adult patients, starting SDHD training or about to start SDHD. In the initial protocol, ICHD patients were matched to SDHD patients, in a 1:1 ratio based on sex, age (±36 months), and duration of dialysis (±1 year). However, most centers had no ICHD patient matching those criteria and the protocol had to be amended. The age-matching window was first expanded to ±5 years but matching remained difficult. Consequently, the protocol was amended once more: for SDHD patients for whom no matched ICHD counterpart could be identified, as well as for subsequently enrolled SDHD patients, one ICHD patient younger than 80 years was to be randomly selected for each SDHD patient. Randomization was stratified by center.

Exclusion criteria included the need for any form of walking assistance (*e.g*., wheelchair, crutches, walker, cane, or similar devices), active malignancy, life expectancy of less than 1 year, substantial reading or writing difficulties, participation in another clinical or interventional study, or a history of major psychiatric disorders or cognitive impairment not adequately managed within the past 5 years.

### Dialysis Treatment and Measure of Physical Activity

Hemodialysis treatment for ICHD consisted of conventional thrice-weekly sessions of approximately 4 hours using the standard hemodialysis machines available in each center. High-flux dialyzers were used at the discretion of treating physicians.

SDHD was carried out with the Physidia S3 monitor, which uses bagged dialysate. SDHD were scheduled five to six sessions per week, lasting 2–3 hours, with a dialysate volume of 20–25 L per session. Dialysate electrolyte composition followed standard prescriptions, determined at the discretion of the treating physician.

To objectively capture physical activity, each participant received a CE-marked Fitbit Flex 2 wristband (provided by the study sponsor). Patients were instructed to wear the device for at least 7 consecutive days during the first week of each calendar month over the 12-month follow-up period. In addition, participants completed the self-administered Kidney Disease Quality of Life questionnaire short form (KDQOL-SF 1.2)^15^ and quantified their recovery time after dialysis upon inclusion, at 6 and 12 months. The patient was to report the days of dialysis during the wearing of the wristband, corresponding body weights the first day of dialysis and physical activity at inclusion and at end of study on the diary they had been handed upon inclusion. These data contributed to a comprehensive evaluation of patient-reported outcomes.

Beyond these study-specific assessments, all aspects of KRT—including treatment choices, monitoring, and follow-up, were left at the discretion of the patients' usual nephrologists. The study did not require any additional clinical procedures, blood samplings, or diagnostic interventions.

### Objectives and End Points

The primary objective of this study was to compare habitual physical activity levels in SDHD patients with those of ICHD patients receiving conventional ICHD thrice weekly. The primary end point of the study was the average daily number of steps assessed at 3, 6, 9, and 12 months of follow-up.

The secondary end points included sleep duration corresponding to the cumulative minutes of sleep recorded by the wristband between 8:00 pm (date of the given day) and 12:00 pm the following day, cumulative sedentary time recorded by the wristband, frequency and duration of hospitalizations during the study period (V0–V12) and occurrence of major adverse cardiac events (MACE).

### Sample Size Calculation

The sample size calculation was based on the hypothesis of detecting a 1000-step/d difference in mean average daily step count between SDHD and ICHD patients, assuming a SD of 2178 steps in both groups. With five repeated measures and a first-order autoregressive covariance structure, the calculation targeted a 5% significance level and 90% statistical power.

As no published data were available on the correlation between repeated measures, various estimates ranging from 0.4 to 0.6 were considered. Assuming a correlation of 0.6, 53 patients per group were required. To account for potential dropouts and allow adjustment for confounding factors, the final target sample size was set at 80 patients in each hemodialysis modality.

### Statistical Analysis

All analyses were performed in accordance with a prespecified Statistical Analysis Plan, which was finalized before database lock and is provided as Supplemental Material. Continuous quantitative variables are summarized by their median, first, and third quartiles. All statistical tests were two sided with a significance of 5%.

Two populations of analysis were defined. The full analysis set (FAS) included all patients who met all inclusion criteria and none of the exclusion criteria. The matched analysis set consisted of the 1:1 matched subset of SDHD and ICHD patients from the FAS.

Patients who discontinued SDHD and resumed ICHD were analyzed within the SDHD group. Because wristband wearing adherence varied across participants, step-count data were extracted within ±42 days of each prespecified time point (90, 180, 270, and 365 days after inclusion), irrespective of the number of days the wristband was worn during each window.

Adverse events and medical history were coded using the MedDRA dictionary (version 27). Missing data were not imputed except for the primary end point. For sensitivity analyses, missing quarterly values of average daily step count were imputed using a last observation carried forward approach limited to data recorded within the same quarter. When no data were available for an entire quarter, no imputation was applied.

The primary end point, average daily step count over time, was analyzed using a generalized linear mixed-effects model for repeated measures, including average daily steps data per month whatever the month may be. Random effects included study center, subject-specific intercepts, and time-related slopes. Fixed-effect covariates included sex, baseline logarithm body mass index (BMI), and chronic heart failure (CHF) and, for the sensitivity analysis in the FAS, also baseline chronologic age. The covariance structure was selected using the lowest Akaike information criterion. Two-way interactions were examined. The same method was used for sleep duration and for sedentary time.

The Kaplan–Meier method was applied to evaluate hemodialysis discontinuation in the SDHD group and MACE. Kidney transplant rates were compared between the two groups using the Gray test, treating death as a competing event. The actuarial method was applied to evaluate hospitalizations. Hospitalization and cardiovascular events rates were compared between the two dialysis groups by using the log-rank test.

The primary analysis was performed using the FAS. The analysis was to be repeated in the matched analysis set if this subset represented <85% of the FAS, serving as a robustness sensitivity analysis.

Analyses were done with SAS version 9.4.

## Results

### Patient Flow and Patient Characteristics

Overall, 28 centers enrolled 152 patients with kidney failure (FAS), including 49 SDHD patients matched with 49 ICHD patients (matched set; Figure 1); overall, 36 patients ended the study prematurely, including 19 patients who received a kidney transplant, three patients who died, three ICHD patients who withdrew their consent, two patients who were lost to follow-up and four patients who moved to another dialysis center, and five patients with no precision on the cause of premature withdrawal. During the study, 14 SDHD patients resumed conventional thrice-weekly ICHD sessions including six SDHD patients of the matched set. At 12 months, the probability to be still treated with SDHD was 68% and its 95% two-sided confidence interval was 56% to 77% (FAS, Supplemental Figure 1). There was no statistical difference in transplant rate between the two dialysis groups (FAS *P* = 0.37, Supplemental Figure 2).

**Figure 1:**
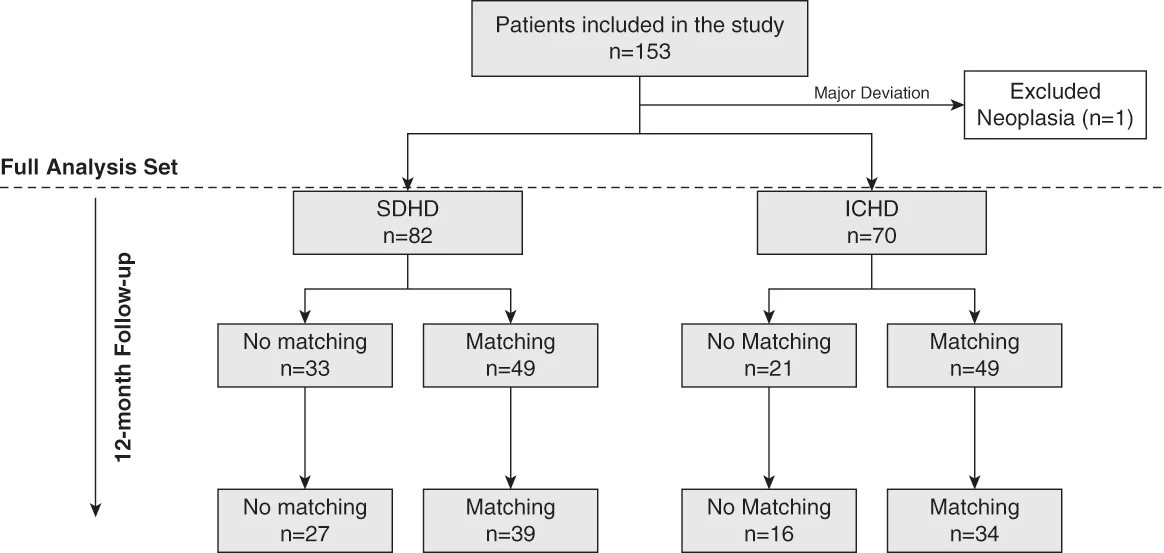
**Patient flow chart.** ICHD, in-center hemodialysis; SDHD, short daily home hemodialysis.

The FAS patient characteristics at baseline are displayed in Table 1. In both groups, most patients were women, older than 40 years and hypertensives. In the matched set, most patients were female (*n*=35 [71%] and *n*=33 [67%] in the SDHD and ICHD groups respectively); the median chronological age was 50 (40–60) and 52 (40–65) years and the median BMI was 25.2 (22.3–28.6) kg/m^2^ and 24.5 (20.8–31.0) kg/m^2^ in the SDHD and ICHD groups, respectively; 44 (90%) and 41 (84%) patients were hypertensive in the SDHD and ICHD groups, respectively.

**Table 1:** Baseline characteristics of the full analysis set including kidney disease and comorbid conditions

Baseline Characteristics	SDHD	ICHD
Number of patients	82	70
Age (yr)	50 (40–64)	56 (41–65)
Sex: Female	55 (67%)	44 (63%)
Postdialysis body weight (kg)	75.3 (66.2–85.1)	70.7 (65.0–88.0)
Height (cm)	173 (164–179)	171 (164–175)
BMI (kg/m^2^)	25.1 (22.2–28.6)	24.6 (20.8–31.6)
**Kidney disease or causal nephropathy**		
Unknown	2 (2%)	7 (10%)
Chronic GN	20 (24%)	15 (21%)
Chronic interstitial nephritis	13 (16%)	2 (3%)
Polycystic kidney disease	13 (16%)	5 (7%)
Diabetes mellitus	11 (13%)	12 (17%)
Hypertensive kidney disease	6 (7%)	9 (13%)
Vascular kidney disease	1 (1%)	3 (4%)
Other	16 (20%)	17 (24%)
Smoking status: Current smoker	25 (31%)	13 (19%)
Hypertension	73 (89%)	59 (84%)
Cardiovascular history	18 (22%)	19 (27%)
Diabetes	16 (20%)	21 (30%)
Dialysis vintage (mo)	0.7 (0.2–1.8)	1.4 (0.7–2.4)
CKD duration (y)	6.5 (3.0–14.0)	8.9 (2.3–15.1)
**Vascular access type**		
Arteriovenous fistula	74 (90%)	58 (83%)
Catheter	8 (10%)	12 (17%)
Distance from home to center	21 (8–37)	8 (4–20)
Prior kidney transplant	15 (18%)	18 (26%)
Restless leg syndrome	10 (12%)	3 (4%)

Data are median (first quartile–third quartile) or *No.* (% column) patients. BMI, body mass index; ICHD, in-center hemodialysis; SDHD, short daily home hemodialysis.

In the FAS, the median average daily step count was 6333 (3579–7631) steps and 4138 (2296–6060) steps in the SDHD and ICHD groups, respectively. In the matched set, the median average daily step count was 6673 (3891–8751) steps and 4758 (2296–6454) steps in the SDHD and ICHD groups, respectively.

Treatment schedules and dialysis prescriptions at baseline in the FAS are presented in Table 2. SDHD was characterized by higher treatment frequency with shorter sessions (120 minutes) at lower blood and dialysate flows/volumes and greater use of buttonhole access and low mol wt heparin, whereas ICHD was associated with higher dialysate potassium concentrations and more frequent use of unfractionated heparin anticoagulation.

**Table 2:** Treatment schedules and dialysis prescriptions at baseline (full analysis set)

Baseline Characteristics	SDHD	ICHD
Number of patients	82	70
Dialysis sessions/wk	6 (5–6)	3 (3–3)
Treatment session duration (min)	120 (120–150)	240 (240–240)
**Vascular access**		
Arteriovenous fistula	74 (90%)	58 (83%)
Catheter	8 (10%)	12 (17%)
**Needling method**		
Missing	8	12
Buttonhole	53 (72%)	15 (26%)
Rope-ladder rotation	21 (28%)	43 (74%)
**Operational conditions**		
Blood flow (ml/min)	300 (270–300)	350 (300–400)
Dialysate flow (ml/min)	180 (180–180)	—
Dialysate Ca^2+^ (mmol/L)	1.5 (1.5–1.5)	1.5 (1.5–1.7)
Dialysate K^+^ (mmol/L)	1.5 (1.0–1.5)	2.0 (2.0–3.0)
Dialysate lactate (mmol/L)	45 (45–45)	—
Dialysate HCO_3_ (mmol/L)	35 (35–35)	34 (32–35)
Dialysate citrate (mmol/L)	—	1 (1–1)
**Anticoagulation**		
None	14 (17%)	2 (3%)
Unfractionated heparin	9 (11%)	22 (31%)
Low molecular weight heparin	59 (72%)	46 (66%)

Data are median (first quartile–third quartile) or *No.* (% column) patients. ICHD, in-center hemodialysis; SDHD, short daily home hemodialysis.

### Primary Outcome

The average number of steps was inversely associated with age and BMI. During the study, patients receiving SDHD had significantly higher mean daily step counts than those receiving ICHD (Figure 2; FAS *P* = 0.008; matched set *P* = 0.03). There was no significant effect of time on step counts (*P* = 0.60) and no significant interaction between dialysis group and time (*P* = 0.32). In the FAS, the difference between groups was no longer statistically significant after adjustment for chronologic age (*P* = 0.08 for dialysis group and *P* = 0.002 for age). In the matched set, however, the difference remained significant after adjustment for CHF and BMI (*P* = 0.02). A significant interaction between CHF and dialysis group was observed for mean daily step count (*P* = 0.10). CHF was reported in five patients (10%) in each group. Across both groups, patients with CHF had lower step counts than those without CHF. Among patients without CHF, SDHD was associated with higher mean daily step counts at baseline and throughout follow-up (Supplemental Figure 3). Data on whether measurements corresponded to dialysis or nondialysis days were insufficient to perform the planned analyses stratified by treatment day or by type of physical activity.

**Figure 2:**
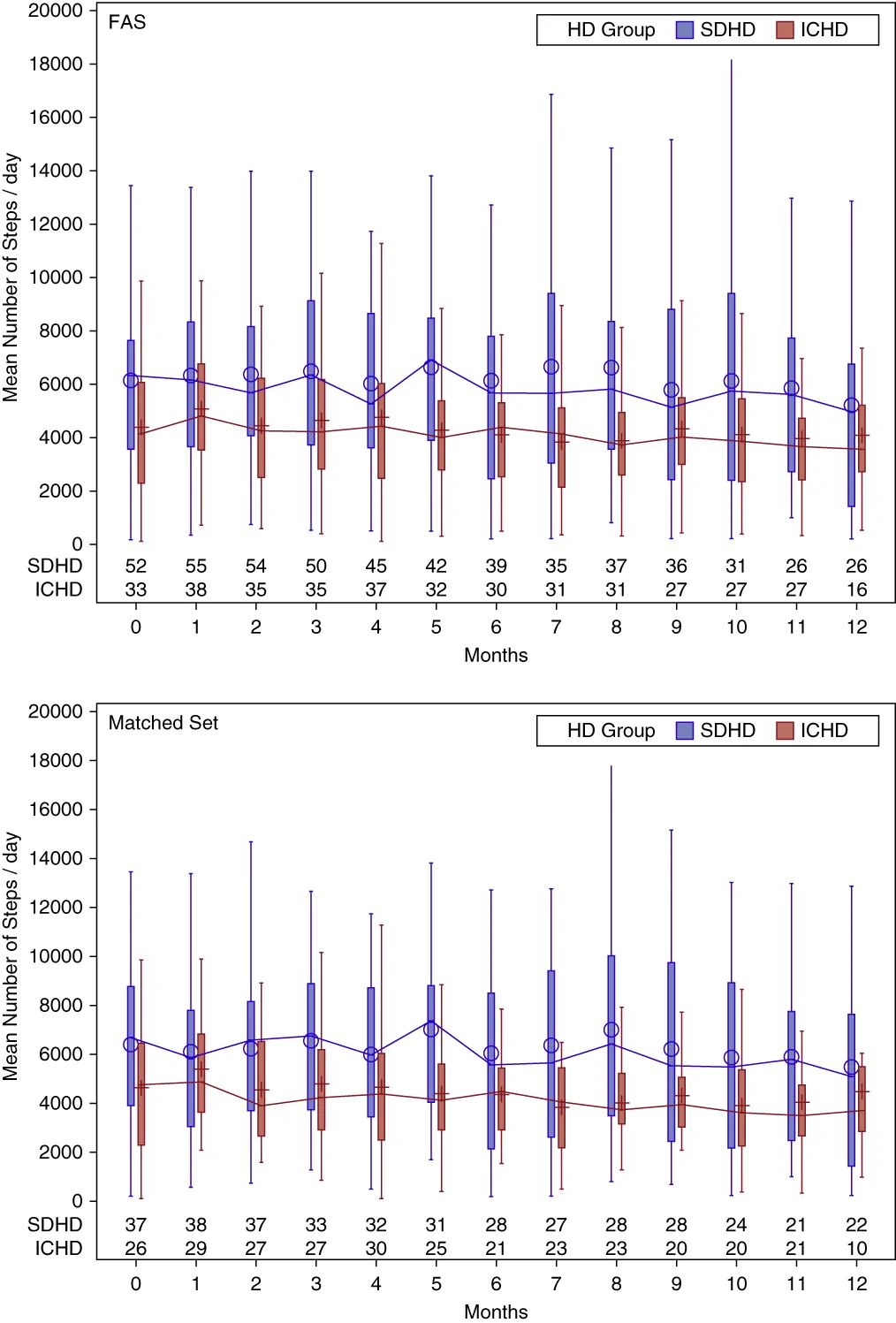
**Average daily step count (steps/d) in the SDHD and ICHD groups over the 12-month follow-up.** Box plots are shown for the FAS (upper panel) and for the matched set (lower panel). Data are presented as medians with IQR for patients with available measurements; lines connect group medians over time. Circles indicate mean values for SDHD, and crosses indicate mean values for ICHD at each time point. Whiskers extend to the most extreme observations within 1.5×IQR (Tukey method). Numbers below the *x*-axis indicate the number of documented patients at each time point. FAS, full analysis set; HD, hemodialysis; IQR, interquartile range.

### Secondary Outcomes

Patients receiving SDHD were more likely to wear the connected wristband at least once than those receiving ICHD (FAS 69 [60%] and 47 [41%; *P* = 0.01); matched set 43 (54%) versus 36 (46%) patients (*P* = 0.08). In the FAS, age (*P* = 0.46), sex (*P* = 0.17), BMI (*P* = 0.43) and hypertension (*P* = 0.88) were not significantly associated with wristband use. At baseline, the median sleep duration was 386 (304–444) versus 380 (323–435) minutes/d in SDHD and ICHD patients, respectively (FAS). Sleep duration tended to be longer in the SDHD group (FAS *P* = 0.06; matched set *P* = 0.05) with a significant interaction between dialysis group and time (FAS *P* = 0.02; matched set *P* = 0.007). Sleep duration was slightly greater over time in the SDHD group but lower in the ICHD group, reaching 405 (345–470) versus 202 (127–296) minutes/d at 12 months, respectively (Figure 3). Sedentary time, already lower in the SDHD group at baseline (FAS 1028 [822–1237] versus 1404 [1221–1440] minutes/d), remained consistently lower throughout follow-up (Figure 4), including after adjustment (FAS *P* = 0.001; matched set *P* = 0.003). Postdialysis recovery time tended to be shorter in SDHD patients (approximately 60 minutes) than in ICHD (approximately 120 minutes; Table 3). KDQOL domain scores tended to be more favorable in the SDHD group, including physical functioning, fatigue, and social participation (Supplemental Table 1). Overall, 32 (39%) and 24 (34%) patients in the FAS were hospitalized during the study (*P* = 0.90). In the matched set, 17 and 18 patients, respectively, were hospitalized (log-rank test *P* = 0.22). Median cumulative hospitalization duration was 7 [2–17] days in the SDHD and 4 [2–11] days in the ICHD group. At least one MACE occurred in 14 and 12 patients in the SDHD and ICHD groups, respectively, in the FAS (log-rank test *P* = 0.74), and in ten patients in each group in the matched set.

**Figure 3:**
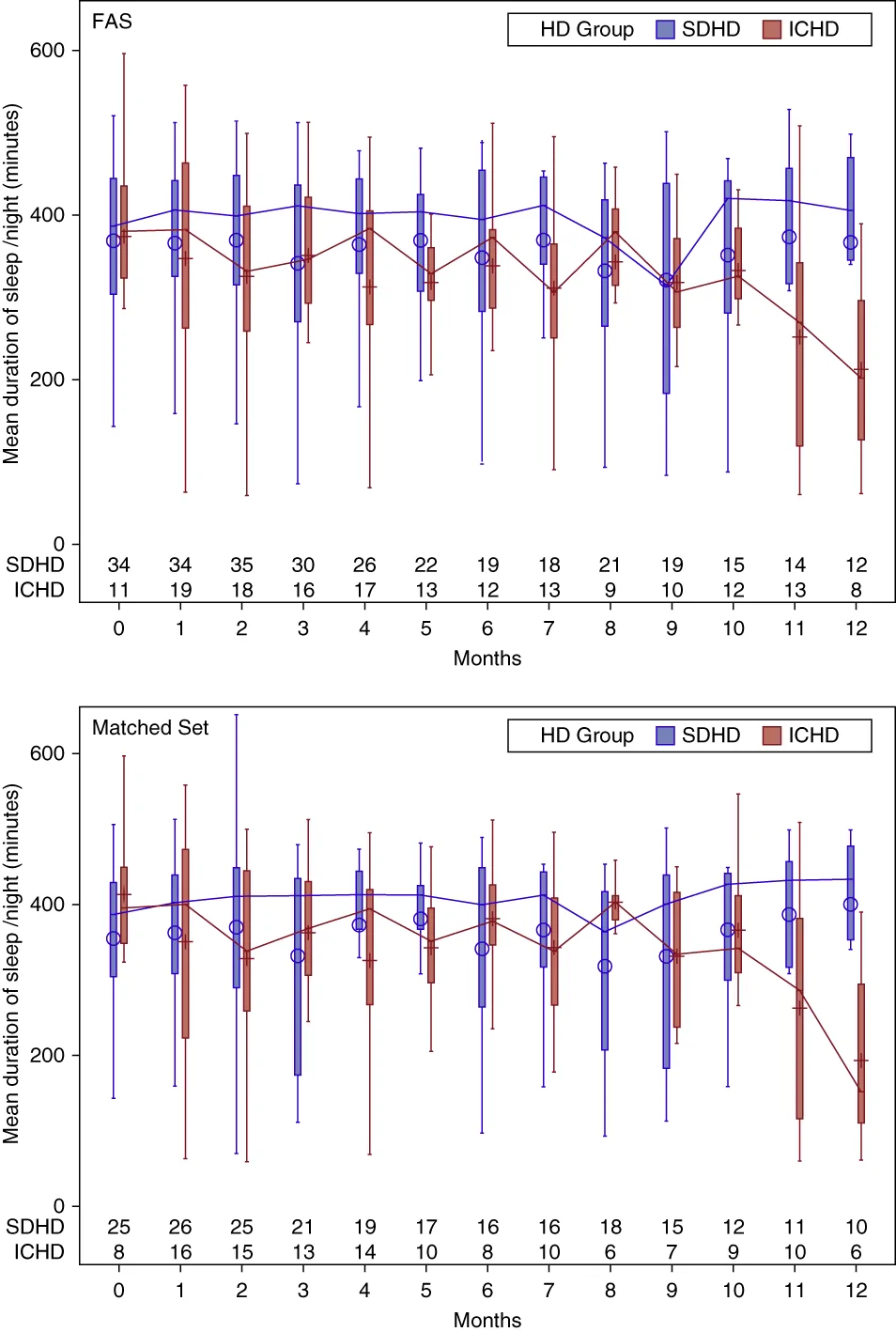
**Average sleep duration (min/night) in the SDHD and ICHD groups over the 12-month follow-up.** Box plots are shown for the FAS (upper panel) and for the matched set (lower panel). Data are presented as medians with IQR for patients with available measurements; lines connect group medians over time. Circles indicate mean values for SDHD, and crosses indicate mean values for ICHD at each time point. Whiskers extend to the most extreme observations within 1.5×IQR (Tukey method). Numbers below the *x*-axis indicate the number of documented patients at each time point.

**Figure 4:**
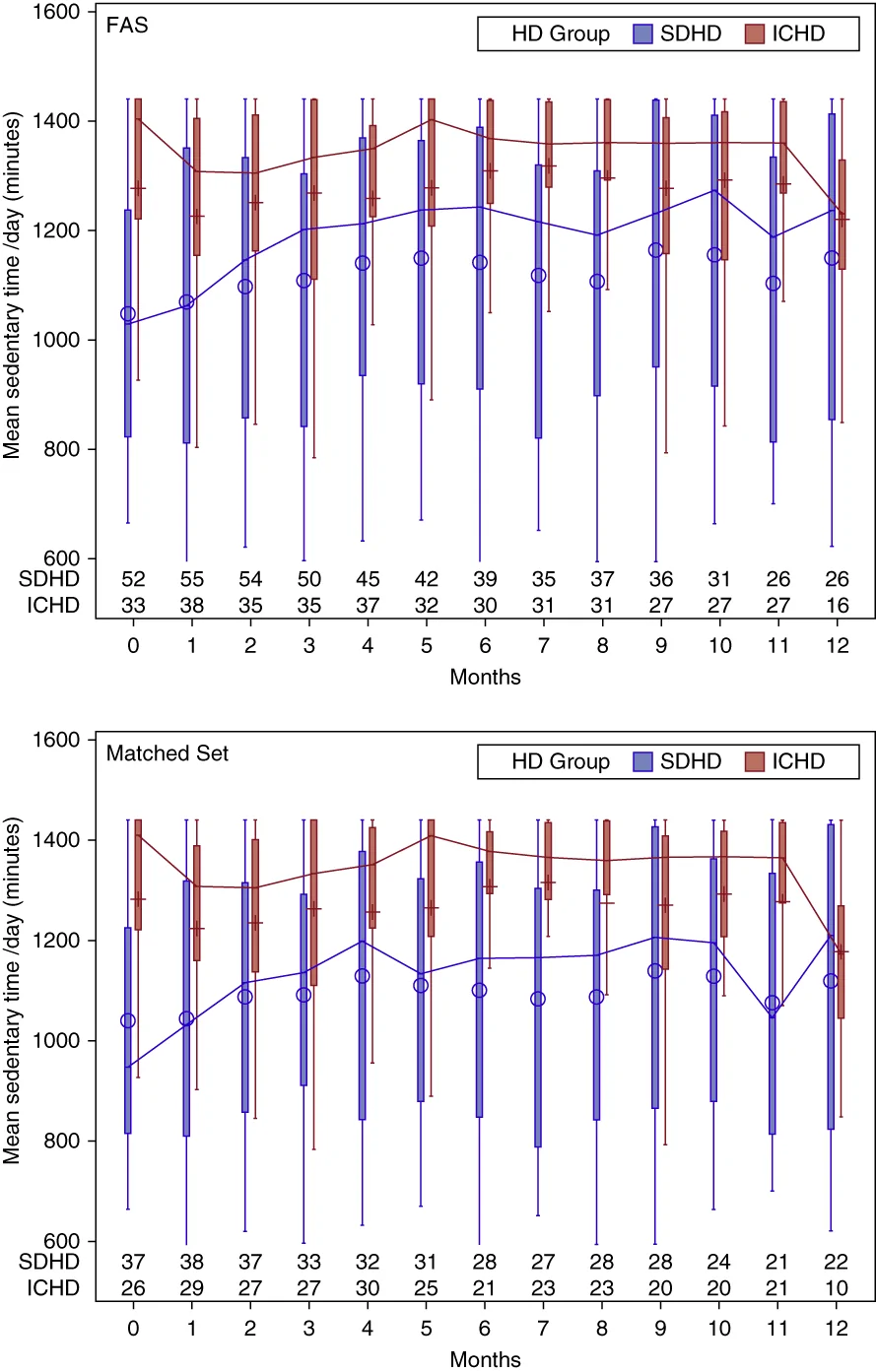
**Average sedentary time (min/24 h) in the SDHD and ICHD groups over the 12-month follow-up.** Box plots are shown for the FAS (upper panel) and for the matched set (lower panel). Data are presented as medians with IQR for patients with available measurements; lines connect group medians over time. Circles indicate mean values for SDHD, and crosses indicate mean values for ICHD at each time point. Whiskers extend to the most extreme observations within 1.5×IQR (Tukey method). Numbers below the *x*-axis indicate the number of documented patients at each time point.

**Table 3:** Dialysis sessions and postdialysis recovery time in the short daily home hemodialysis and in-center hemodialysis groups over the 12-mo follow-up

Baseline Characteristics	Inclusion		6 Mo		12 Mo	
	SDHD	ICHD	SDHD	ICHD	SDHD	ICHD
**FAS**						
Number hemodialysis sessions/wk						
*Missing*	0	0	17	9	2	3
*3*	1 (1%)	69 (99%)	7 (12%)	46 (92%)	11 (18%)	45 (96%)
*5*	35 (43%)	—	17 (30%)	0 (0%)	16 (26%)	0 (0%)
*6*	45 (55%)	—	31 (54%)	3 (6%)	33 (53%)	2 (4%)
*Other*	1 (1%)	1 (1%)	2 (4%)	1 (2%)	2 (3%)	0 (0%)
Duration of hemodialysis sessions (min)						
*n*	82	70	57	50	62	47
*Median (first quartile–third quartile)*	120 (120–150)	240 (240–240)	120 (120–150)	240 (240–240)	120 (120–150)	240 (240–240)
Recovery time (min)						
*n*	61	51	31	26	34	25
*Median (first quartile–third quartile)*	60 (30–240)	120 (30–480)	60 (15–120)	120 (30–180)	30 (5–60)	90 (15–240)
**Matched set**						
Number hemodialysis sessions/wk						
*Missing*	0	0	9	9	1	2
*3*	1 (2%)	49 (100%)	4 (12%)	29 (91%)	7 (19%)	31 (97%)
*5*	24 (49%)	—	13 (38%)	0 (0%)	10 (27%)	0 (0%)
*6*	24 (49%)	—	16 (47%)	3 (9%)	19 (51%)	1 (3%)
*Other*	—	—	1 (3%)	0 (0%)	1 (3%)	0 (0%)
Duration of hemodialysis sessions (min)						
*n*	49	49	57	50	62	47
*Median (first quartile–third quartile)*	120 (120–150)	240 (240–240)	120 (120–150)	240 (240–240)	120 (120–150)	240 (240–240)
Recovery time (min)						
*n*	39	39	18	18	25	18
*Median (first quartile–third quartile)*	90 (30–240)	120 (30–360)	60 (1–120)	120 (30–240)	60 (5–60)	105 (15–240)

Data are shown for the FAS and, where applicable, for the matched set. Categorical variables are presented as *n* (%), and continuous variables as median (first quartile–third quartile). Three sessions per week in the SDHD group correspond to the conventional ICHD regimen. The patient in the SDHD group receiving three sessions per week at inclusion had just transitioned to SDHD at study inclusion. Missing indicates patients without documented data for the corresponding variable. FAS, full analysis set; ICHD, in-center hemodialysis; n, number of patients with documented data; SDHD, short home daily hemodialysis.

### Additional Analyses

SDHD patients consistently maintained higher predialysis and postdialysis body weights (Supplemental Figure 4 and Supplemental Table 2). BP was generally similar between modalities (Supplemental Table 2). SDHD patients tended to have lower predialysis *β*2-microglobulin concentrations (Figure 5). A summary of other laboratory parameters in the FAS is provided in Supplemental Table 3. Electrolytes (Na^+^, HCO_3_^−^) remained stable and comparable between groups. Serum phosphate and calcium were well controlled in both groups. Bone metabolism markers (alkaline phosphatase, parathyroid hormone) showed wide variability. Hemoglobin levels were similar across groups (≈10.5–11.5 g/dl), although transferrin saturation and ferritin levels tended to be lower in SDHD. Serum albumin levels were slightly higher in SDHD (≈40–42 versus 38–40 g/L in ICHD).

**Figure 5:**
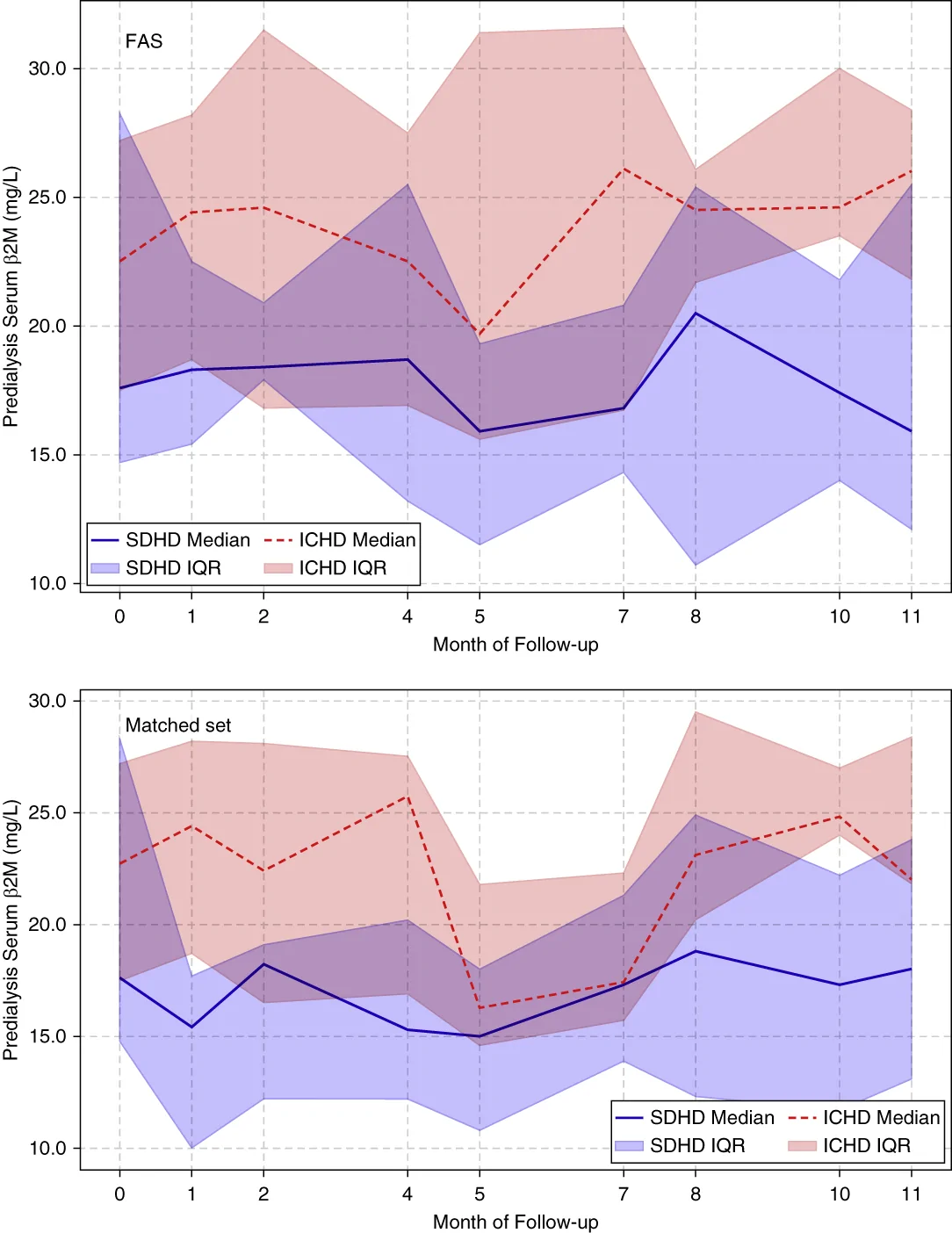
**Predialysis serum *β*2-microglobulin concentrations (mg/L) in the SDHD and ICHD groups over the 12-month follow-up.** Data are shown for the FAS (upper panel) and the matched set (lower panel). Values are presented as medians with IQR for patients with available measurements, and lines connect group medians over time.

Prescriptions of erythropoiesis-stimulating agents were comparable between groups. However, over the 12-month follow-up, SDHD tended to receive fewer antihypertensive agents and fewer phosphate binders than ICHD patients (Supplemental Table 4).

## Discussion

The applied matching strategy resulted in patient groups with comparable baseline characteristics, including age, body mass, and BMI. This study compared the clinical evolution of patients with kidney failure treated with SDHD versus conventional thrice-weekly ICHD over 12 months. The primary objective was to assess physical activity, whereas secondary end points included dialysis efficacy, treatment tolerance, acceptability, anemia and nutritional management, and patient-reported outcomes.

A notable finding is that physical activity tended to be better preserved in patients treated with SDHD. At baseline, SDHD patients achieved a 28% higher daily step count than ICHD patients (6673 versus 4768 steps/d), a difference that remained stable throughout follow-up despite the natural decline in activity over time. Importantly, SDHD patients maintained similar step counts on dialysis and nondialysis days, whereas ICHD patients exhibited marked reductions in activity on treatment days. This pattern underscores the disruptive impact of conventional ICHD schedules on daily functioning, whereas SDHD appears to provide a more physiological treatment rhythm that better integrates into daily life. Beyond activity measures, SDHD was associated with several clinical and patient-centered benefits. Sedentary time (Figure 4) was substantially lower, sleep duration was maintained rather than reduced, and postdialysis recovery time was shorter compared with ICHD, findings that were associated with better hemodynamic tolerance and a faster return to baseline functioning. Health-related QoL scores were also better preserved across several domains, while treatment burden appeared less pronounced, with higher adherence and technical survival. Furthermore, a greater proportion of SDHD patients were waitlisted for transplantation and subsequently transplanted, suggesting enhanced transplant eligibility. Although hospitalizations were fewer in the SDHD group and cardiovascular events were not higher, statistical power was limited by the small number of events.

Treatment efficacy and metabolic control were also favorable in the SDHD cohort. Predialysis *β*2-microglobulin concentrations were consistently lower, suggesting superior middle-molecule clearance,^16^ a surrogate marker linked with a reduced risk of cardiovascular event and systemic inflammation.^17–20^ BP control was broadly comparable between modalities, with SDHD patients likely benefiting from greater vascular stability and reduced hemodialysis-induced stress. Mineral metabolism parameters (phosphate, calcium, parathyroid hormone, alkaline phosphatase) were similarly controlled in both groups, indicating that SDHD prescriptions are safe and effective for bone-mineral management. Anemia management was comparable in terms of hemoglobin levels; however, SDHD patients exhibited lower ferritin and transferrin saturation despite similar hemoglobin targets. This was associated with lower iron exposure, potentially reflecting lower inflammation, better nutritional status, and a reduced requirement for intravenous iron therapy despite more frequent dialysis sessions and potentially greater blood losses. Nutritional status, as reflected by higher body weight and serum albumin, was slightly better in SDHD patients, consistent with the less restrictive fluid and dietary allowances permitted by more frequent dialysis.

These findings are consistent with previous randomized trials and registry-based analyses.^21–24^ More frequent and intensive dialysis treatment schedules have been associated with improvements in laboratory parameters and cardiovascular parameters and may reduce mortality. However, the impact on patient-reported outcomes and QoL, appears more heterogeneous and depends on the treatment modality and schedule (*e.g*., nocturnal or extended versus daily dialysis) as well as patient characteristics. The Following Rehabilitation, Economics and Everyday-Dialysis Outcome Measurements (FREEDOM) study provided real-world evidence that daily home hemodialysis improves QoL, postdialysis recovery time, cognitive performance, depression scores, and hospitalization burden.^25–27^ Randomized controlled trials from the frequent hemodialysis network^28^ and the Canadian nocturnal trial^29^ demonstrated that more frequent hemodialysis was associated with favorable effects on cardiac structure, including regression of left ventricular hypertrophy, along with improvements in patient reported outcomes such as physical health and recovery time. However, these benefits were accompanied by an higher risk of vascular access–related complications.^28^ Real-world registry data also support a survival benefit of home dialysis, potentially mediated by better nutritional status and reduced inflammation.^23,24,30–34^ The observation of lower ferritin and reduced iron use in patients in SDHD patients aligns with reports suggesting that home hemodialysis is associated with a less proinflammatory milieu, thereby reducing the need for iron supplementation and mitigating long-term risks of iron overload.

The strengths of this study include its prospective longitudinal design, systematic monitoring of both biologic and functional parameters, and direct comparison of SDHD with conventional ICHD treatment. Several limitations should be acknowledged. The nonrandomized design introduces the potential for selection bias because SDHD patients are often younger and more motivated. Attrition reduced sample sizes over time; and treatment prescriptions (dialysis dose, eryhtropoetin stimulating agent, and iron protocols) were not standardized, which may have contributed to intergroup differences.

The study highlights that SDHD provides not only equivalent but, in several domains, superior outcomes relative to conventional ICHD. By minimizing treatment-related disruption to daily activity, shortening recovery time, and enhancing middle molecule clearance. SDHD appears to promote a better patient experience, preserve physical activity, and offer more physiological dialysis profile. These findings support the broader implementation of SDHD programs as a safe and effective alternative to in-center dialysis. Future research should include larger prospective studies assessing long-term survival, cardiovascular outcomes, and cost-effectiveness, as well as optimized anemia and iron management strategies tailored to home-based regimens.

Short daily home hemodialysis was associated with preserved physical activity, shorter post-dialysis recovery time, enhanced middle-molecule clearance, and effective anemia and nutritional management, while maintaining target dialysis adequacy. Collectively, these findings support SDHD as a patient-centered modality with potential clinical and QoL benefits, warranting broader evaluation and further investigation.

## Supplementary Material

## Data Availability

Original data generated for the study will be made available upon reasonable request to the corresponding author. Data Type: Aggregated Data. Reason for Restricted Access: The data underlying this study are not publicly available due to patient confidentiality and data protection regulations. De-identified data may be made available from the corresponding author upon reasonable request, subject to institutional approval and data use agreements.

## Declarative Statements

This study includes clinical experimentation and received Institutional Review Board or Ethics Committee approval. All patients provided written informed consent. This study includes clinical experimentation and complies with the Declaration of Helsinki. The clinical and research activities being reported are consistent with the Principles of the Declaration of Istanbul as outlined in the Declaration of Istanbul on Organ Trafficking and Transplant Tourism.

## Supplemental Material

Supplemental Methods. Statistical analysis plan.

Supplemental Figure 1. Discontinuation of Short-Daily Home Hemodialysis (FAS).

Supplemental Figure 2. Kidney transplant (FAS).

Supplemental Figure 3. Mean average daily steps in patients with no CHF.

Supplemental Figure 4. Body weight after dialysis.

Supplemental Table 1. KDQOL scores (FAS).

Supplemental Table 2. Predialysis weight and BP during the study (FAS).

Supplemental Table 3. Blood laboratory data (FAS).

Supplemental Table 4. Medications (FAS).

## References

[1] Bello AK Okpechi IG Osman MA, . Epidemiology of haemodialysis outcomes. Nat Rev Nephrol. 2022;18(6):378–395. doi:10.1038/s41581-022-00542-7 PMC8862002 35194215

[2] See E Ethier I Cho Y, . Dialysis outcomes across countries and regions: a global perspective from the international society of nephrology global kidney health atlas study. Kidney Int Rep. 2024;9(8):2410–2419. doi:10.1016/j.ekir.2024.05.014 PMC11328589 39156158

[3] Li PK Chan GC Chen J, . Tackling dialysis burden around the world: a global challenge. Kidney Dis (Basel). 2021;7(3):167–175. doi:10.1159/000515541 PMC8215964 34179112

[4] Rostoker G Dumas JJ Thome B Aragno VA Thomas M Canaud B. Cost-effectiveness of daily home haemodialysis versus in-centre haemodialysis: a propensity score matching analysis based on real-world data from the French healthcare system. Eur J Intern Med. 2025;137:73–82. doi:10.1016/j.ejim.2025.04.017. 40318912

[5] Vanholder R Davenport A Hannedouche T, .; Dialysis Advisory Group of American Society of Nephrology. Reimbursement of dialysis: a comparison of seven countries. J Am Soc Nephrol. 2012;23(8):1291–1298. doi:10.1681/ASN.2011111094 22677554

[6] Torreggiani M Fois A Chatrenet A, . Incremental and personalized hemodialysis start: a new standard of care. Kidney Int Rep. 2022;7(5):1049–1061. doi:10.1016/j.ekir.2022.02.010 PMC9091804 35571001

[7] Prichard SS. Comorbidities and their impact on outcome in patients with end-stage renal disease. Kidney Int. 2000;57(S74):S100–S104. doi:10.1046/j.1523-1755.2000.07417.x

[8] Blankestijn PJ Vernooij RWM Hockham C, .; CONVINCE Scientific Committee Investigators. Effect of hemodiafiltration or hemodialysis on mortality in kidney failure. N Engl J Med. 2023;389(8):700–709. doi:10.1056/NEJMoa2304820 37326323

[9] Rose M Fischer FH Liegl G, .; CONVINCE Scientific Committee and CONVINCE Investigators. The CONVINCE randomized trial found positive effects on quality of life for patients with chronic kidney disease treated with hemodiafiltration. Kidney Int. 2024;106(5):961–971. doi:10.1016/j.kint.2024.07.014 39089577

[10] Vernooij RWM Hockham C Barth C, . High-target hemodiafiltration convective dose achieved in most patients in a 6-month intermediary analysis of the CONVINCE randomized controlled trial. Kidney Int Rep. 2023;8(11):2276–2283. doi:10.1016/j.ekir.2023.08.004 PMC10658200 38025213

[11] Vernooij RWM Hockham C Strippoli G, . Haemodiafiltration versus haemodialysis for kidney failure: an individual patient data meta-analysis of randomised controlled trials. Lancet. 2024;S0140-6736(24)01859-2. doi:10.1016/S0140-6736(24)01859-2 39489903

[12] Fessi H Nicoud P Serrato T, . Two years' experience of intensive home hemodialysis with the physidia S(3) system: results from the RECAP study. J Clin Med. 2023;12(4):1357. doi:10.3390/jcm12041357 PMC9958970 36835894

[13] Fessi H Szelag JC Courivaud C, . Safety and efficacy of short daily hemodialysis with physidia S(3) system: clinical performance assessment during the training period. J Clin Med. 2022;11(8):2123. doi:10.3390/jcm11082123 PMC9031690 35456216

[14] Target N Courivaud C Michel PA Daoud S Thomas M. Comparison of physical activity and quality of life in home haemodialysis (HHD) patients versus conventional in-centre haemodialysis (ICHD) patients: the observational, longitudinal, prospective, international, multicentric SeCoIA study protocol. BMC Nephrol. 2020;21(1):500. doi:10.1186/s12882-020-02127-7 PMC7682091 33225917

[15] Hays RD Kallich JD Mapes DL Coons SJ Amin N Cart WB, editors. Kidney Disease Quality of Life Short Form (KDQOL-ST), Version 1.3: A Manual for Use and Scoring. RAND Corporation; 1997.

[16] Perrin-Haarhoff A Henri P Cerasuolo D, . Efficacy of slow daily home hemodialysis with internal convection on removal of uremic toxins using the Physidia S3 monitor. Sci Rep. 2025;15(1):11609. doi:10.1038/s41598-025-91985-y PMC11971319 40185794

[17] Cheung AK Rocco MV Yan G, . Serum β2-microglobulin levels predict mortality in dialysis patients: results of the HEMO study. J Am Soc Nephrol. 2006;17(2):546–555. doi:10.1681/ASN.2005020132 16382021

[18] Maduell F Moreso F Pons M, ESHOL Study Group. High-efficiency postdilution online hemodiafiltration reduces all-cause mortality in hemodialysis patients. J Am Soc Nephrol. 2013;24(3):487–497. doi:10.1681/ASN.2012080875 PMC3582206 23411788

[19] Kanda E Masakane I Kerr PG, . Beta-2 microglobulin and all-cause mortality in the era of high-flux hemodialysis: results from the dialysis outcomes and practice patterns study. Clin Kidney J. 2020;14(5):1436–1445. doi:10.1093/ckj/sfaa155 PMC8087125 33959272

[20] Okuno S Ishimura E Kohno K, . Serum beta2-microglobulin level is a significant predictor of mortality in maintenance haemodialysis patients. Nephrol Dial Transplant. 2009;24(2):571–577. doi:10.1093/ndt/gfn521 18799606

[21] Bonenkamp AA van Eck van der Sluijs A Hoekstra T, . Health-related quality of life in home dialysis patients compared to in-center hemodialysis patients: a systematic review and meta-analysis. Kidney Med. 2020;2(2):139–154. doi:10.1016/j.xkme.2019.11.005 PMC7380444 32734235

[22] Budhram B Sinclair A Komenda P Severn M Sood MM, . A Comparison of patient-reported outcome measures of quality of life by dialysis modality in the treatment of kidney failure: a systematic review. Can J Kidney Health Dis. 2020;7:2054358120957431. doi:10.1177/2054358120957431 PMC7580133 33149924

[23] Tennankore KK Kim SJ Baer HJ Chan CT. Survival and hospitalization for intensive home hemodialysis compared with kidney transplantation. J Am Soc Nephrol. 2014;25(9):2113–2120. doi:10.1681/ASN.2013111180 PMC4147990 24854268

[24] Tennankore KK Na Y Wald R Chan CT Perl J. Short daily-nocturnal- and conventional-home hemodialysis have similar patient and treatment survival. Kidney Int. 2018;93(1):188–194. doi:10.1016/j.kint.2017.06.014 28844317

[25] Jaber BL Finkelstein FO Glickman JD, FREEDOM Study Group. Scope and design of the following rehabilitation, economics and everyday-dialysis outcome measurements (FREEDOM) Study. Am J Kidney Dis. 2009;53(2):310–320. doi:10.1053/j.ajkd.2008.07.013 18823688

[26] Jaber BL Lee Y Collins AJ, FREEDOM Study Group. Effect of daily hemodialysis on depressive symptoms and postdialysis recovery time: interim report from the FREEDOM study. Am J Kidney Dis. 2010;56(3):531–539. doi:10.1053/j.ajkd.2010.04.019 20673601

[27] Jaber BL Schiller B Burkart JM, FREEDOM Study Group. Impact of short daily hemodialysis on restless legs symptoms and sleep disturbances. Clin J Am Soc Nephrol. 2011;6(5):1049–1056. doi:10.2215/CJN.10451110 PMC3087770 21415315

[28] Chertow GM Levin NW Beck GJ, .; FHN Trial Group. In-center hemodialysis six times per week versus three times per week. N Engl J Med. 2010;363(24):2287–2300. doi:10.1056/NEJMoa1001593 PMC3042140 21091062

[29] Culleton BF Walsh M Klarenbach SW, . Effect of frequent nocturnal hemodialysis vs conventional hemodialysis on left ventricular mass and quality of life: a randomized controlled trial. JAMA. 2007;298(11):1291–1299. doi:10.1001/jama.298.11.1291 17878421

[30] Cheetham MS Ethier I Krishnasamy R, . Home versus in-centre haemodialysis for people with kidney failure. Cochrane Database Syst Rev. 2024;4(4):CD009535. doi:10.1002/14651858.CD009535.pub3 PMC11001293 38588450

[31] Ishani A Greer N Taylor BC, . Comparative Effectiveness of Home-based Kidney Dialysis Versus in-center Dialysis: A Systematic Review and meta-analysis. Department of Veterans Affairs; 2015. 26937543

[32] Marshall MR Polkinghorne KR Boudville N McDonald SP. Home versus facility dialysis and mortality in Australia and New Zealand. Am J Kidney Dis. 2021;78(6):826–836. doi:10.1053/j.ajkd.2021.03.018 33992726

[33] Dubrofsky L Ibrahim A Tennankore K Poinen K Shah S Silver SA. An environmental scan and evaluation of home dialysis quality indicators currently used in Canada. Can J Kidney Health Dis. 2020;7:2054358120977391. doi:10.1177/2054358120977391 PMC7734484 33354332

[34] Tennankore KK Chan CT Curran SP. Intensive home haemodialysis: benefits and barriers. Nat Rev Nephrol. 2012;8(9):515–522. doi:10.1038/nrneph.2012.145 22825665

