# Supplementary material for: Physical Activity in Short Daily Home Dialysis versus to In-Center Hemodialysis: Results from the Self-convecTive Evaluation of Short Daily Home Dialysis on Physical Activity Study
Source: Kidney360. 2026 Jun 18;7(9):2075–86. doi: 10.34067/KID.0000001246 (PMC13641458; doi:10.34067/KID.0000001246)
Supplement: Supplementary file 1 [file kidney360-7-2075-s001.pdf]

ASN
American Society of Nephrology
JASN
Journal of the American Society of Nephrology
KIDNEY360
Accessing Our World From Every Angle
CJASN
Clinical Journal of the American Society of Nephrology

ASN Journal Disclosure Form

As per ASN journal policy, I have disclosed any financial relationships or commitments I have held in the past 36 months as included below. I have listed my Current Employer below to indicate there is a relationship requiring disclosure. If no relationship exists, my Current Employer is not listed.

B. Canaud reports the following:
Employer: MTX Consulting Int; and Other Interests or Relationships: CEO of MTX Consulting Int, Montpellier-France.

I understand that the information above will be published within the journal article, if accepted, and that failure to comply and/or to accurately and completely report the potential financial conflicts of interest could lead to the following: 1) Prior to publication, article rejection, or 2) Post-publication, sanctions ranging from, but not limited to, issuing a correction, reporting the inaccurate information to the authors' institution, banning authors from submitting work to ASN journals for varying lengths of time, and/or retraction of the published work.

Name: Bernard J. Canaud
Manuscript ID: K360-2026-000191R1
Manuscript Title: Physical Activity in Short Daily Home Hemodialysis versus In Center Hemodialysis: Results from fhe STEP-HD Study
Date of Completion: April 15, 2026
Disclosure Updated Date: April 15, 2026

ASN American Society of Nephrology | JASN Journal of the American Society of Nephrology | KIDNEY360 Accessing Our World From Every Angle | CJASN Clinical Journal of the American Society of Nephrology

ASN Journal Disclosure Form

As per ASN journal policy, I have disclosed any financial relationships or commitments I have held in the past 36 months as included below. I have listed my Current Employer below to indicate there is a relationship requiring disclosure. If no relationship exists, my Current Employer is not listed.

C. Courivaud has nothing to disclose.

I understand that the information above will be published within the journal article, if accepted, and that failure to comply and/or to accurately and completely report the potential financial conflicts of interest could lead to the following: 1) Prior to publication, article rejection, or 2) Post-publication, sanctions ranging from, but not limited to, issuing a correction, reporting the inaccurate information to the authors' institution, banning authors from submitting work to ASN journals for varying lengths of time, and/or retraction of the published work.

Name: Cécile Courivaud
Manuscript ID: K360-2026-000191R2
Manuscript Title: Employer This field defaults to the Organization field in your ASN profile. If you or your spouse/partner have been employed by any for-profit company, currently or during the past 24 months, please list that here also.
Date of Completion: June 1, 2026
Disclosure Updated Date: June 1, 2026

ASN American Society of Nephrology · JASN Journal of the American Society of Nephrology · KIDNEY360 Accessing Our World From Every Angle · CJASN Clinical Journal of the American Society of Nephrology

ASN Journal Disclosure Form

As per ASN journal policy, I have disclosed any financial relationships or commitments I have held in the past 36 months as included below. I have listed my Current Employer below to indicate there is a relationship requiring disclosure. If no relationship exists, my Current Employer is not listed.

F. Le Floc'h reports the following:
Employer: Monitoring Force Group

I understand that the information above will be published within the journal article, if accepted, and that failure to comply and/or to accurately and completely report the potential financial conflicts of interest could lead to the following: 1) Prior to publication, article rejection, or 2) Post-publication, sanctions ranging from, but not limited to, issuing a correction, reporting the inaccurate information to the authors' institution, banning authors from submitting work to ASN journals for varying lengths of time, and/or retraction of the published work.

Name: Fabien Le Floc'h
Manuscript ID: K360-2026-000191R1
Manuscript Title: Physical Activity in Short Daily Home Hemodialysis versus In Center Hemodialysis: Results from fhe STEP-HD Study
Date of Completion: May 4, 2026
Disclosure Updated Date: April 22, 2026

ASN American Society of Nephrology | JASN Journal of the American Society of Nephrology | KIDNEY360 Accessing Our World From Every Angle | CJASN Clinical Journal of the American Society of Nephrology

ASN Journal Disclosure Form

As per ASN journal policy, I have disclosed any financial relationships or commitments I have held in the past 36 months as included below. I have listed my Current Employer below to indicate there is a relationship requiring disclosure. If no relationship exists, my Current Employer is not listed.

P. Michel reports the following:
Employer: AP-HP; Honoraria: CSL Vifor; and Speakers Bureau: CSL Vifor.

I understand that the information above will be published within the journal article, if accepted, and that failure to comply and/or to accurately and completely report the potential financial conflicts of interest could lead to the following: 1) Prior to publication, article rejection, or 2) Post-publication, sanctions ranging from, but not limited to, issuing a correction, reporting the inaccurate information to the authors' institution, banning authors from submitting work to ASN journals for varying lengths of time, and/or retraction of the published work.

Name: Pierre Antoine Michel
Manuscript ID: K360-2026-000191R1
Manuscript Title: Physical Activity in Short Daily Home Hemodialysis versus In Center Hemodialysis: Results from fhe STEP-HD Study
Date of Completion: May 5, 2026
Disclosure Updated Date: May 5, 2026

ASN American Society of Nephrology
JASN Journal of the American Society of Nephrology
KIDNEY360 Accessing Our World From Every Angle
CJASN Clinical Journal of the American Society of Nephrology

ASN Journal Disclosure Form

As per ASN journal policy, I have disclosed any financial relationships or commitments I have held in the past 36 months as included below. I have listed my Current Employer below to indicate there is a relationship requiring disclosure. If no relationship exists, my Current Employer is not listed.

N. Target has nothing to disclose.

I understand that the information above will be published within the journal article, if accepted, and that failure to comply and/or to accurately and completely report the potential financial conflicts of interest could lead to the following: 1) Prior to publication, article rejection, or 2) Post-publication, sanctions ranging from, but not limited to, issuing a correction, reporting the inaccurate information to the authors' institution, banning authors from submitting work to ASN journals for varying lengths of time, and/or retraction of the published work.

Name: Natalia Target
Manuscript ID: K360-2026-000191R1
Manuscript Title: Physical Activity in Short Daily Home Hemodialysis versus In Center Hemodialysis: Results from fhe STEP-HD Study
Date of Completion: May 4, 2026
Disclosure Updated Date: May 4, 2026

ASN
American Society of Nephrology
JASN
Journal of the American Society of Nephrology
KIDNEY360
Accessing Our World From Every Angle
CJASN
Clinical Journal of the American Society of Nephrology

ASN Journal Disclosure Form

As per ASN journal policy, I have disclosed any financial relationships or commitments I have held in the past 36 months as included below. I have listed my Current Employer below to indicate there is a relationship requiring disclosure. If no relationship exists, my Current Employer is not listed.

M. Thomas reports the following:
Employer: Physidia

I understand that the information above will be published within the journal article, if accepted, and that failure to comply and/or to accurately and completely report the potential financial conflicts of interest could lead to the following: 1) Prior to publication, article rejection, or 2) Post-publication, sanctions ranging from, but not limited to, issuing a correction, reporting the inaccurate information to the authors' institution, banning authors from submitting work to ASN journals for varying lengths of time, and/or retraction of the published work.

Name: Michel Thomas
Manuscript ID: K360-2026-000191R1
Manuscript Title: Physical Activity in Short Daily Home Hemodialysis versus In-Center Hemodialysis: Results from STEP-HD Study
Date of Completion: May 4, 2026
Disclosure Updated Date: April 15, 2026
