# Supplementary material for: Physical Activity in Short Daily Home Dialysis versus to In-Center Hemodialysis: Results from the Self-convecTive Evaluation of Short Daily Home Dialysis on Physical Activity Study
Source: Kidney360. 2026 Jun 18;7(9):2075–86. doi: 10.34067/KID.0000001246 (PMC13641458; doi:10.34067/KID.0000001246)
Supplement: Supplementary file 2 [file kidney360-7-2075-s002.pdf]

Physical Activity in Short Daily Home Dialysis vs to In-Center HD: Results from the STEP-HD

Step-HD (SeCoIA) Interventional Study

This document is the English translation of the original French version

STATISTICAL ANALYSIS PLAN

Version No. 3.0 dated 28 February 2024

Final

CRO

MONITORING FORCE GROUP

38 rue Jean Mermoz, Bât. D

78600 Maisons-Laffitte, France

Tel: +33 (0) 1 39 62 15 27

Author: Bernadette DARNE

Date and signature

SPONSOR

PHYSIDIA

11 Boulevard des Bretonnières

49124 Saint Barthélémy D'Anjou, France

Tel: +33 (0) 6 74 28 71 99

Medical Director: Michel THOMAS

Coordinating Investigator: Dr Natalia TARGET

Date and signature

TABLE OF CONTENTS

1. LIST OF REFERENCES ABBREVIATIONS ... 5
2. SAP VERSION AND AMENDMENT ... 6
3. STUDY SYNOPSIS ... 6
4. STUDY OBJECTIVES ... 13
4.1. PRIMARY OBJECTIVE ... 13
4.2. SECONDARY OBJECTIVES ... 13
4.3. MODIFICATION OF THE PRIMARY OBJECTIVE DURING THE CONDUCT OF THE STUDY ... 14
5. STUDY DESIGN ... 14
5.1. GENERAL OUTLINE AND EXPERIMENTAL DESIGN ... 14
5.2. RANDOMISATION ... 14
5.3. CALCULATION OF THE NUMBER OF SUBJECTS ... 14
5.4. SOURCE DOCUMENTS ... 15
5.5. STUDY SCHEDULE ... 15
6. STUDY POPULATION ... 16
6.1. SUBJECTS DISPOSITION ... 16
6.2. DEFINITION OF POPULATIONS OF ANALYSIS ... 17
6.2.1 PATIENT POPULATION ... 17
6.2.2 EXPOSED-UNEXPOSED POPULATION ... 17
6.2.3 VIGILANCE POPULATION ... 17
6.2.4 ACTIVE CENTRE POPULATION ... 17
6.3. TREATMENT ASSIGNMENT ERRORS ... 17
6.4. OTHER ANALYSIS POPULATION ... 18
6.5. MAJOR PROTOCOL DEVIATIONS ... 18
6.6. DEFINITION OF ANALYSIS SUBGROUPS ... 18
7. VARIABLES OF ANALYSIS ... 18
7.1. CHARACTERISTICS OF THE SITES ... 18
7.2. DEMOGRAPHIC CHARACTERISTICS AND INCLUSION DATA ... 19
7.2.1 SOCIO-DEMOGRAPHIC DATA ... 19
7.2.2 MEDICAL HISTORY ... 19
7.2.3 COMORBIDITIES ... 20
7.2.4 CONCOMITANT TREATMENTS ... 20
7.2.5 CLINICAL EXAMINATIONS AND LABORATORY DATA ... 21
7.3. MAIN CRITERION ... 21
7.4. SECONDARY CRITERIA ... 21
7.4.1 PHYSICAL ACTIVITY ... 21
7.4.2 DIALYSIS CHARACTERISTICS ... 23
7.4.3 CLINICAL DATA ... 24

7.4.4 HOSPITALISATIONS AND OTHER COMPLICATIONS 25
7.4.5 SELF-ADMINISTERED QUESTIONNAIRES 26
7.5. VIGILANCE PARAMETERS 27
7.5.1 ADVERSE EVENTS 27
7.5.2 VITAL SIGNS AND PHYSICAL EXAMINATIONS 27
7.5.3 LABORATORY PARAMETERS 27
7.6. OTHER PARAMETERS 27
7.6.1 PHYSICAL ACTIVITY AND CONFINEMENT 27
7.6.2 CHARACTERISTICS OF FOLLOW-UP VISITS 28
7.6.3 QUARTERLY GENERAL INFORMATION DATA (V3, V6, V9 AND V12): 28
7.6.4 PREMATURE WITHDRAWAL FROM THE STUDY 28
7.7. COVARIATES 28
8. STATISTICAL ANALYSES 29
8.1. GENERAL 29
8.2. HYPOTHESES TO BE TESTED 30
8.3. GROUPING OF SITES 30
8.4. INTERIM ANALYSES 30
8.5. TIME POINTS FOR ANALYSIS 30
8.5.1 TIME POINT-VISIT 30
8.5.2 TIME POINT FOR WRISTBAND 31
8.5.3 CALCULATION OF TIME LIMITS AND CENSORING 31
8.6. METHOD FOR REPLACING MISSING DATA 31
8.7. CHARACTERISTICS OF THE SITES 31
9. EVALUATION OF DEMOGRAPHIC AND INCLUSION CHARACTERISTICS 31
9.1. DEMOGRAPHIC DATA 32
9.2. MEDICAL HISTORY 32
9.1. CONCOMITANT TREATMENTS (INCLUSION AND FOLLOW-UP) 32
9.2. CLINICAL EXAMINATIONS AND LABORATORY DATA 32
9.3. DIALYSIS CHARACTERISTICS 32
10. ANALYSIS OF EVALUATION CRITERIA 32
10.1. ANALYSIS OF THE MAIN CRITERION 32
10.2. ANALYSIS OF SECONDARY ENDPOINTS 33
10.2.1 PHYSICAL ACTIVITY 33
10.2.2 DIALYSIS CHARACTERISTICS 33
10.2.3 CLINICAL DATA 34
10.2.4 HOSPITALISATIONS AND OTHER COMPLICATIONS 34
10.2.5 SELF-ADMINISTERED QUESTIONNAIRES 35
11. ANALYSIS OF VIGILANCE PARAMETERS 35
11.1. ADVERSE EVENT 35

11.2. VITAL SIGNS AND CLINICAL EXAMINATIONS 35
11.3. LABORATORY PARAMETERS 35
11.4. OTHER ANALYSES 36
11.4.1 PHYSICAL ACTIVITY AND LOCKDOWN 36
11.4.2 CHARACTERISTICS OF FOLLOW-UP VISITS 36
11.4.3 PREMATURE WITHDRAWAL FROM THE STUDY 36
12. RISK ASSESSMENT 36
13. ANALYSIS TABLES AND FIGURES 37
14. REFERENCES 42

1. LIST OF REFERENCES ABBREVIATIONS

ANSM	French National Agency for Safety of Drugs and Health Products (Agence Nationale de Sécurité du Médicament et des produits de santé
ATC	Anatomical Therapeutic Chemical
CNIL	National Commission for Information Technology and Civil Liberties (Commission Nationale de l'informatique et des Libertés)
CNOM	National Medical Council (Conseil National de l'Ordre des Médecins)
CPP	Committee for the Protection of Individuals (Comité de Protection des Personnes)
CRF	Case Report Form
CSP	Public Health Code (Code de la santé Publique)
EI	Adverse Effect
SAE	Serious Adverse Effect
ENE	Exposed – Not Exposed
HCC	Conventional centre-based haemodialysis
HD	Haemodialysis
HDF	Haemodiafiltration
HLGT	High Level Group Term
HHD	Home Haemodialysis
CRF	Chronic Renal Failure
ESKD	End-stage renal disease
IRLS	International Restless Legs Syndrome Rating Scale
KDQOL	Kidney Disease Quality of Life Instrument
Km	Kilometre
MFG	Monitoring Force Group
DBP	Diastolic Blood Pressure
PAS	Statistical Analysis Plan
SBP	Systolic Blood Pressure
PSQI	Pittsburgh Sleep Quality Index
SAS	Statistical Analysis Software®
RLS	Restless Legs Syndrome
SOC	System Organ Class
UAD	Autodialysis Unit (Unité d'Autodialyse)
UDM	Medicalised Dialysis Unit (Unité de Dialyse Médicalisée)

2. SAP VERSION AND AMENDMENT

This document is version 3.0 of the Statistical Analysis Plan (SAP) for the SeCoIA/Step-HD study, based on the amended study protocol, version 9.0 dated 10/05/2021.

This SAP details the methods for the final analysis of the data.

This version of the SAP includes the second amendment to the SAP.

Amendment 1: The objective of this amendment is as follows:

- To remove the interim analysis planned in the protocol: Due to the SARS-CoV-2 epidemic, which has caused a significant delay in monitoring visits and the resolution of correction requests, thereby shifting the study's provisional schedule, the sponsor has decided to remove the interim analysis planned in the protocol (see section 8.4).

  In the study protocol, analysis of the primary endpoint is only planned for the final analysis. Therefore, the removal of the interim analysis has no impact on the results of the analysis.
- Modify the time windows of follow-up visits (see section 8.5.1).

Amendment 2: The objective of this amendment is as follows:

- Specify the characteristics defining a valid Fitbit wearing: eliminate small values that could correspond to simple transport or testing of the wristband (section 7.3).
- Specify the methods for calculating derived variables: body mass index (section 7.2.1) and duration of ESKD and dialysis (section 7.2.2).

Note: All text extracted from the protocol is in *italics*, except for the study summary, to facilitate reading.

3. STUDY SYNOPSIS

**Study title**	Efficiency of Home Haemodialysis (HHD) compared to conventional centre-based haemodialysis (HCC): SeCoIA Interventional Study - Step-HD
**Rationale**	The FHN study and the FREEDOM study are the two main studies that have examined the effects of daily dialysis in a centre or at home compared to three weekly dialysis in a centre, and provide useful information on the medical benefits and quality of life. These two studies, like most studies on this subject, are American studies. French data on daily home dialysis are scarce and cover only a small number of patients.<br><br>The data in the literature confirm that daily home haemodialysis would allow for more effective dialysis and improve patients' quality of life, with fewer complications and hospitalisations than dialysis three times a week. However, this technique remains little used in France and the number of discontinuations is relatively high, due in particular to the possibility for patients to receive kidney transplant, but not only.<br><br>In addition, few studies have evaluated the physical activity of dialysis patients, and those that have been conducted have mainly involved patients haemodialysed in centres and a small number of subjects. One of these studies, conducted in a centre in the United Kingdom, also included patients on daily home haemodialysis, but with a small number of subjects, 28 in total.<br><br>The aim of this study is to describe the characteristics of patients on daily home haemodialysis (HHD) and to confirm in real-world practice the efficacy shown in

	studies, mainly American, both in terms of medical outcomes and quality of life. Due to the limited number of studies available in this population, particular emphasis will be placed on physical activity.<br>This study is exploratory, and the most appropriate type of study appears to be an exposed (HHD) / unexposed (centre-based haemodialysis, HCC) study.
**Objectives**	Main objective:<br>The primary objective of this study is to evaluate physical activity in the daily lives of HHD patients compared to HCC patients undergoing treatment three times a week.<br>Secondary objectives:<br>The secondary objectives are as follows:<br>1. To compare physical activity among HCC subjects between dialysis days and non-dialysis days;<br>2. Describe the characteristics of HHD patients;<br>3. To describe prescribed modalities of HHD;<br>4. Compare blood pressure trends between the two haemodialysis modalities;<br>5. Compare the frequency of hospitalisations, their duration and the hospital departments involved between the two haemodialysis modalities;<br>6. Compare the incidence of cardiovascular complications between the two haemodialysis modalities;<br>7. Compare the quality of life of patients undergoing the two types of haemodialysis;<br>8. Assess the quality of patients' sleep between HHD and HCC;<br>9. Compare the percentage of patients with restless legs syndrome (RLS) and the severity of RLS between HHD and HCC;<br>10. Compare changes in medication use between HHD and HCC;<br>11. Assess the number of subjects who discontinue HHD and the reasons for discontinuation;<br>12. Compare patients who use pedometers/accelerometers with those who do not, based on some parameters: age, gender, body size, blood pressure.
**Primary endpoints**	**Primary endpoint:**<br>The primary endpoint is the total average number of steps taken per day at 3 months, 6 months, 9 months and 12 months.<br>**Secondary criteria:**<br>1. Average number of steps taken per day by HCC patients on days without dialysis and days with dialysis;<br>2. Patient characteristics;<br>3. Haemodialysis modalities for HHD patients;<br>4. Changes in blood pressure;<br>5. Hospitalisations, duration and departments;<br>6. Cardiovascular complications;<br>7. KDQOL 1.2 questionnaire (Kidney Disease Quality of Life Instrument);<br>8. Average sleep duration and PSQI (Pittsburg Sleep Quality Index) questionnaire;<br>9. Percentage of patients with RLS and IRLS (International Restless Legs Syndrome Rating Scale) questionnaire;<br>10. Drug treatments;<br>11. Percentage of patients having discontinued HD at 12 months and reasons for discontinuation..

**Study design**	This is an interventional, pragmatic, minimal risk and constraint, exposed/unexposed, prospective, longitudinal, multicentre study conducted in Belgium and mainland France.<br>The intervention comprises the provision of:<br>• A connected wristband (pedometer/accelerometer), which patients are instructed to wear for a minimum of seven consecutive days each calendar month.<br>• Self-administered questionnaires, to be completed by the patient.<br>• A patient diary.<br>The connected bracelet that will be used for the study is the FITBIT®Flex 2™ wristband provided by the sponsor to all participating patients. This wristband has CE marking (see compliance commitment in Appendix 6 of the protocol).<br>The decision to offer HHD, as well as the associated conditions for this modality and for HCC, forms part of the standard management of end-stage chronic kidney disease as determined by the treatment centre and the responsible physician. Participation in the study is only proposed once the patient's hemodialysis modality (HHD or HCC) has already been established. Consequently, no hemodialysis equipment is supplied as part of this study.<br>In this study, the exposed subjects are HHD patients and the unexposed subjects are HCC patients undergoing treatment three times a week [haemodialysis (HD) or haemodiafiltration (HDF)].<br>Given the difficulties of matching, it has been decided to eliminate matching and to randomly select the unexposed subjects from among those aged < 80 years, undergoing haemodialysis in a centre three times a week and followed by a study physician. The random selection will be stratified by centre (a centre is defined by all the physicians participating in the study at that centre).<br>An ancillary medico-economic study may be carried out using certain data collected in this study (hospitalisations, cardiovascular complications, dialysis-related complications, surgeries, drug treatments, medical transport consumption, etc.) in order to determine costs and, if necessary, by collecting additional data. This ancillary study will be the subject of a separate protocol.
**Duration of the study**	• Total duration of the study: 45 months<br>○ Recruitment: 33 months<br>○ Follow-up: 12 months
**Selection of centres**	The centres are haemodialysis centres, medical dialysis units (UDM) or self-dialysis units (UAD) that have prescribed daily home dialysis at least once.<br>The study will be offered to all centres identified by the sponsor as practicing home haemodialysis.
**Number of centres**	Approximately 40 centres are expected to agree to participate in the study.
**Patient recruitment**	Each centre must include at least the first two patients for whom HHD is prescribed on the day or no later than 15 days after the HHD prescription. For each new HHD patient, a haemodialysis patient treated at the centre and monitored by one of the physicians participating in the study at the HHD patient's centre will be randomly selected.<br>Recruitment is competitive between centres.

**Patient inclusion criteria**	1. Male or female aged 18 years or older;<br>2. Patient who has dated and signed the consent form;<br>3. Patient affiliated with a Social Security scheme<br>4. Exposed subjects: patients who are starting or about to start daily haemodialysis training (5, 6 or 7 times per week) at home using haemodialysis techniques.<br>5. Non-exposed subjects: prevalent or incident patients undergoing dialysis at a centre (3 times per week) selected at random. Preferably, the random selection will be carried out on subjects in UAD or UDM.
**Patient exclusion criteria**	1. The patient requires any type of assistance to walk (wheelchair, crutch, walker, cane, etc.).<br>2. Active neoplasia or neoplasia undergoing treatment<br>3. Life expectancy of less than one year<br>4. Patient with significant difficulties reading or writing<br>5. Patient participating in a clinical trial or other interventional study<br>6. History of mental instability, major cognitive disorders in the previous 5 years, or major psychiatric disorder that is not adequately controlled or stable under pharmacological treatment.
**Practical procedure**	Before starting patients' enrollment, the physician will complete the centre questionnaire.<br>Before inclusion in the study, the investigator will explain the objectives of the study and inform the patient of his/her rights, both verbally and in writing. Each patient must sign an informed consent form.<br>Upon enrollment in the study, the patient will receive a connected wristband, to be worn on the arm opposite the vascular access for at least seven consecutive days each month (at inclusion and subsequently during the first week of each calendar month), from waking until bedtime at a minimum.<br>Patients will also be given a "patient diary" to complete at the start of the study and then the first week of each calendar month.<br>The centres including patients will follow them in the study for 12 months after their inclusion.<br>Home HHD and HCC treatments and patient follow-up will be carried out in accordance with the centre's usual practices.<br>Only consultation data and test results available as part of the patient's usual follow-up will be collected for the study. All data will be collected during follow-up visits scheduled by physicians as part of their routine practice.<br>Self-administered quality of life and sleep questionnaires will be completed by the patient and given to the doctor during routine care visits at inclusion, at 6 months and at the end of the study.<br>Data available from the connected wristband will be collected at inclusion and then every month.<br>Data will be collected from four categories of documents: a centre questionnaire, a case report form (CRF) with duplicate sheets, patient self-questionnaires and a patient diary.

	The data will be collected on paper, with the exception of data from the connected wristband, which will be collected in electronic format after anonymisation.
**Data collected**	**<u>Centre questionnaire:</u>**<br>The following data will be collected on the "centre questionnaire":<br>○ Centre department<br>○ Type of centre<br>○ Number of prevalent patients undergoing HDD<br>○ Year of 1st prescription of HDD<br><br>**<u>Data reported in the observation log:</u>**<br>1. Data collected during the inclusion visit<br>a) Preliminary data:<br>○ Date of informed consent signature;<br>○ Verification of inclusion and exclusion criteria;<br>○ Date of the inclusion visit.<br>b) Socio-demographic data:<br>○ Age;<br>○ Sex;<br>○ Height;<br>○ Pre- and post-dialysis weight from the last dialysis session before the visit + consultation weight;<br>○ Smoking status;<br>○ Employment status;<br>○ Transportation option for coming to the centre;<br>○ Distance between home and centre.<br>c) Blood pressure: before/after dialysis session (collection of the last 3 available measurements, on 3 different days).<br>d) Characteristics of the chronic kidney disease (CKD):<br>○ Pathology causing CKD;<br>○ Date of diagnosis;<br>○ Date of initiation of haemodialysis;<br>○ Previous transplant(s);<br>○ Transplant status;<br>○ Comorbidities;<br>○ Presence of SDJSR.<br>e) Current drug treatments (name of treatment, dose, unit, route and frequency). Current drug treatments will be recorded on the "Treatments" form in the CRF.<br>f) Dialysis characteristics:<br>○ Dialysis location;<br>○ Number of sessions per week;<br>○ Dialysis duration;<br>○ Type of vascular access;<br>○ Type of needle;<br>○ Type of cannulation technique;<br>○ Needle size;<br>○ Anticoagulants;<br>○ Blood flow;<br>○ Dialysate composition.<br>➢ Data collected only from patients undergoing dialysis at a centre:<br>○ Dialysis method;

	- Total convective volume per session for HDF patients;<br>- Plasma volume control technique.<br>➢ Data collected only from HHD patients:<br>- Start date of training;<br>- Prescribed convective volume per session;<br>- Dialysate flow rate.<br><br>g) Relevant biological data (latest available blood sample): haemoglobin, glycated haemoglobin, calcium, phosphorus, PTH, bicarbonates, alkaline phosphatases, ferritin, transferrin saturation coefficient, albumin, sodium, pre-dialysis β2-microglobulin level.<br>h) Urine test with collection method (sample, 12-hour or 24-hour urine collection): creatininuria, urea, proteinuria, microalbuminuria.<br>i) Connected wristband: wristband number given to the patient, arm on which the wristband is worn.<br><br>2. Data collected during monthly visits (V1, V2, V4, V5, V7, V8, V10, V11):<br><br>*a)* Date of visit, if performed<br><br>*b)* Patient status: continuing haemodialysis at the centre/continuing haemodialysis at home/stopping haemodialysis at home and returning to the centre/discontinuation of haemodialysis.<br><br>*c)* Clinical data:<br>- Pre- and post-dialysis weight from the last dialysis session before the visit + consultation weight;<br>- Presence of RLS.<br><br>*d)* Blood pressure: before/after dialysis in the week preceding the visit (collection of the last 3 available measurements, on 3 different days)<br><br>*e)* Dialysis characteristics (collected only for patients on HHD):<br>- Number of weekly sessions,<br>- Usual duration of dialysis.<br><br>*f)* Hospitalisations and complications:<br>- Hospitalisations: reasons, duration, hospital department(s) (if surgery: PMSI category);<br>- Complications related to dialysis;<br>- Compilation of adverse events.<br><br>*g)* Changes in current medication (discontinued, modified, initiated) since the last visit.<br><br>*h)* Relevant biological data (see V0)<br><br>*i)* Urine test (see V0)<br><br>j) Connected wristband:<br>- Wearing of the bracelet since the last consultation;<br>- If synchronisation is performed.<br><br>3. Data collected during quarterly visits (V3, V6, V9 and V12):<br><br>*a)* General information:<br>- Employment status;

	○ Transportation option for coming to the centre;<br>○ Distance between home and centre.<br><br>*b)* Data from a monthly visit (see monthly visit)<br>a) Dialysis characteristics (see V0)<br><br>4. Data collected on the study exit form:<br>○ Date of last visit or last telephone contact;<br>○ Premature exit (yes/no);<br>○ Reasons for study exit in case of premature exit.<br><br>5. Data collected on the haemodialysis or daily dialysis discontinuation form:<br>○ Date of last dialysis session;<br>○ Reason for discontinuation;<br>○ Data collected only for patients discontinuing HHD: planned return to centre: yes/no.<br><br>**Data collected from the connected wristband:**<br>The data collected from the connected wristband are:<br>○ Number of steps;<br>○ Activity times;<br>○ Sleep.<br><br>**Data collected from the patient diary:**<br>The data collected from the patient diary are:<br>○ Start and end dates for wearing the wristband;<br>○ Dialysis days;<br>○ Weight before and after dialysis on the first day of dialysis during the period.<br>○ Physical activity and details thereof upon inclusion in the study<br>○ Physical activity and details thereof upon completion of the study<br>○ Patient assessment of their activity at the end of the study compared to when they were included in the study<br><br>**Other collected data:**<br>• In order to collect data on patient recovery time, the following question will be added to the KDQOL quality of life questionnaire: 'When your dialysis session ends, how long does it take for you to feel like you're back to your usual state?'<br>• In order to assess patients' physical activity during the lockdown linked to the Covid-19 epidemic, a specific questionnaire was given to the patients.
**Justification of sample size**	Two publications were selected to estimate the number of subjects needed[1,2] . The number of subjects needed was estimated using PASS® software to estimate the odds ratio of a continuous quantitative parameter with matching. The standard deviation of the total number of steps used for the estimate is that of haemodialysis patients in Carvalho *et* al[22]: 2178 steps/day. With a 5% α risk , a two-tailed test, one unexposed subject for one exposed subject, the number of evaluable subjects needed to demonstrate an odds ratio of 1.2 or 1.3 for an increase of 1,000 varies between 65 and 80 subjects per group, depending on whether the power is 80% or 90%.<br><br>It has been decided to include 80 exposed subjects and 80 unexposed subjects, i.e. 160 subjects in the study, which will provide sufficient power even if the number of evaluable subjects is only 130.

	The number of subjects is not changed by the amendment. The analysis will be adjusted for confounding factors.
**Statistical methods**	The statistical analysis will be performed using SAS version 9.4 and SAS/STAT15.1 or later (SAS Institute, North Carolina, USA).<br>A detailed statistical analysis plan will be drafted and validated by the sponsor before the database is frozen and the statistical analysis begins.<br>The parameters will be described in the usual way:<br>• Continuous quantitative variables (e.g. age, weight, height, BP) will be described by the frequency of documented data, the frequency of missing values, the mean, the standard deviation, the 1st and 3rd quartiles and the extreme values.<br>• Categorical variables (e.g. sex) will be described by the frequency and percentage of each category. The frequency of missing data will also be displayed.<br>Statistical tests will be two-tailed with a statistical significance threshold of 5%.<br>Unless otherwise specified, the comparison of exposed and unexposed subjects will be performed using a generalised linear mixed model. The analysis will be adjusted for the following parameters: sex, age of subjects, length of haemodialysis, body mass index, diabetes mellitus, high blood pressure and heart failure.<br>The mean total number of steps will be analysed as a repeated measure (inclusion, 3, 6, 9 and 12 months) in the logistic regression model.<br>The dropout rate at 12 months will be estimated in subjects on HHD using the Kaplan-Meier method. The number and percentage of subjects who underwent transplantation during the study period will be presented in the two groups.<br>The rate and time to 1st hospitalisation will be estimated using the Kaplan-Meier method, and exposed and unexposed subjects will be compared using a Cox model adjusted for the parameters listed above. Cardiovascular complications will be analysed in the same way as 1st hospitalisation.<br>An interim analysis will be performed on all subjects included for at least 6 months as of 31 March 2021. The interim analysis will only be performed on secondary endpoints and therefore does not change the number of subjects needed.
**Provisional timetable**	○ Regulatory submission in France: May 2018<br>○ Establishment of centres: September 2018 to January 2020<br>○ Regulatory submission in Belgium: November 2019<br>○ Patient enrolment period: September 2018 – June 2021<br>○ End of patient follow-up: June 2022<br>○ Interim analysis: Q4 2021<br>○ Database closure and final analysis: Q4 2022

4. STUDY OBJECTIVES

4.1. Primary objective

The primary objective of this study is to assess habitual physical activity in patients undergoing HHD and to compare it with that of patients receiving in-centre conventional haemodialysis (HCC) three times per week.

4.2. Secondary Objectives

The secondary objectives are:

1. *To compare physical activity of CKD patients between dialysis days and non-dialysis days;*
2. *To describe the characteristics of patients on HHD;*
3. *To describe the prescription procedures of HHD;*
4. *Compare blood pressure trends between HHD and HCC;*
5. *Compare the frequency of hospitalisations, their duration and hospital departments between HHD and HCC;*
6. *Compare the incidence of cardiovascular events between HHD and HCC;*
7. *Compare the quality of life of patients between HHD and HCC;*
8. *Assess the quality of patients' sleep between HHD and HCC;*
9. *Compare the percentage of patients with restless legs syndrome (RLS) and the severity of RLS between HHD and HCC;*
10. *Compare changes in medication use (iron, erythropoietin, antihypertensives, phosphate binders, vitamin D, calcium supplements, calcimimetics, bicarbonates, calcium carbonates, potassium chelators, oral anticoagulants, heparin, hypercholesterolemia treatments, diabetes treatments, painkillers, medical treatments for RLS and sleep disorders) between between HHD and HCC;*
11. *Assess the number of subjects who discontinue HHD and the reasons for discontinuation;*
12. *Compare patients who use pedometers/accelerometers with those who do not with respect to demographic and clinical characteristics, including age, sex, body size, blood pressure.*

4.3. Modification of the primary objective during the conduct of the study

None.

5. STUDY DESIGN

5.1. General outline and experimental design

This is an interventional, pragmatic, minimal risk and constraint, exposed/unexposed, prospective, longitudinal, multicentre and national study conducted in Belgium and mainland France.

5.2. Randomisation

There is no randomisation of subjects in this study, but a random draw of unexposed subjects (HCC) when the initially planned matching was not possible.

The random draw will be carried out with stratification by centre. The number of unexposed subjects (HCC) to be included for each centre will correspond to the number of HHD subjects included and without a matched unexposed subject. For example, if a centre included two HHD subjects for whom no HCC subjects could be matched at the time of implementation of Amendment 3 to the protocol*, then two HCC subjects selected by random draw must be included. Similarly, if a centre included 2 HHD patients after the implementation of amendment 3, then 2 HCC subjects selected at random must be included by one of the doctors participating in the study at that centre.*

To avoid excessive imbalance between the groups, only HCC subjects under the age of 80 at the time of inclusion may be included in the study.

No treatment randomisation or blinding was performed in this study.

5.3. Calculation of the number of subjects

Two publications were selected for estimating the number of subjects needed[11,2]. The number of subjects required was estimated using PASS® software to estimate the odds ratio of a continuous quantitative parameter with matching. The standard deviation of the total number of steps used for the estimate is that of subjects on haemodialysis in Carvalho et al[2 2] : 2,178 steps/day. With a 5% α risk, a two-tailed test, one unexposed subject for one exposed subject, the number of evaluable subjects

needed to show an odds ratio of 1.2 or 1.3 for an increase of 1,000 steps varies between 65 and 80 subjects per group, depending on whether the power is 80% or 90%.

It was decided to include 80 exposed subjects and 80 unexposed subjects, i.e. 160 subjects in the study, which will provide sufficient power even if the number of evaluable subjects is only 130.

The removal of matching changes the analysis methods. With a repeated measures analysis comparing two means and the hypotheses of a difference in the mean number of 1,000 steps per day[1] with a standard deviation of 2,178 steps[2], identical in both groups, five measurements and a first-order autoregressive variance-covariance matrix structure and the same assumptions as above and a power of 90%, the number of subjects depends on the correlation of the number of steps between two measurement times for a given subject. In the absence of reference data, the estimate was made with different levels of correlation. If the correlation is 0.4, 38 HHD subjects and the same number of HCC subjects are required; if the correlation is 0.6, then 53 HHD subjects and 53 HCC subjects are required. These numbers do not take into account the number of non-evaluable subjects, and the power will depend on the number of missing values.

It was therefore decided not to change the number of subjects required, which will also allow the analysis to be adjusted for confounding factors.

5.4. Source documents

The parameters analysed will be those from the centre questionnaire, the CRF, the self-questionnaires, the patient diary and those extracted from the connected wristband.

The study data are collected on the following questionnaires:

- Patient data:
    - CRF
    - Self-questionnaires completed by patients:
        - KDQOL
        - IRLS for patients with RLS
        - Physical activity questionnaire during lockdown
    - Patient diary
- Site data:
    - Site questionnaire

With the exception of wristband data, data is entered into a database (electronic CRF) after completion of paper forms by investigators or patients.

The variable names are available in the annotated eCRF version 1.0 dated 05/11/2020.

Wristband data are available in electronic format in a separate file.

5.5. Study schedule

The provisional schedule for the study is as follows:

- *Regulatory submission in France: May 2018*
- *Set-up of centres: September 2018 to January 2020*
- *Regulatory submission in Belgium: November 2019*
- *Patient enrolment period: September 2018 – June 2021*
- *End of patient follow-up: June 2022*
- *Database closure and final analysis: Q4 2022*

The updated timeline is as follows: the interim analysis will be performed in Q4 2022, the final database will be frozen in Q1 2023, and the final analysis will be performed in Q2 2023.

6. STUDY POPULATION

The study will include a population of HCC patients and HHD patients who meet the inclusion criteria and none of the exclusion criteria.

It is planned to recruit 160 patients into the study (80 exposed subjects and 80 unexposed subjects).

The patient selection criteria are as follows:

Inclusion criteria

To be eligible for inclusion in the study, participants must meet all of the following criteria:

1. *Male or female aged 18 years or older;*
2. *Patient who has dated and signed the consent form;*
3. *Patient enrolled in a social security scheme;*
4. *Exposed subjects: patients who are starting or about to start training on daily haemodialysis (5, 6 or 7 times a week) at home using haemodialysis techniques;*
5. *Non-exposed subjects: patients under the age of 80 who are currently undergoing or will undergo dialysis at a centre (3 times per week), selected at random*

   Preferably, randomisation will be carried out on subjects in self-dialysis units (UAD) or medical dialysis units (UDM).

Inclusion criterion No. 5 was modified in the 3rd amendment to the protocol dated 23/07/2020. Due to matching difficulties, it was decided to no longer match unexposed subjects to exposed subjects, but to randomly select a sample of unexposed subjects.

Non-inclusion criteria

To be included, the patient must meet none of the following exclusion criteria:

1. *Needs for any form of walking assistance (e.g., wheelchair, crutches, walker, cane, or similar devices)*
2. *Active neoplasia or current treatment of neoplasia*
3. *Life expectancy of less than one year*
4. *Participant with significant reading or writing difficulties*
5. *Current participation in a clinical trial or other interventional study;*
6. *History of mental instability, major cognitive disorders in the previous 5 years, or major psychiatric disorder not adequately controlled or stable under pharmacological treatment.*

Notably, refusal to wear the pedometer or accelerometer does not constitute an exclusion criterion, nor does a return to haemodialysis following transplant failure.

6.1. Subjects Disposition

The disposition of subjects, as indicated below, will be presented by exposure group and overall:

- Total number of patients included in the study,
- Total number of patients included in the different populations of analysis,
- Frequency and percentage of patients with major protocol deviations, by type of deviation,
- Frequency and percentage of patients with premature discontinuation,
- Frequency and percentage of reasons for premature discontinuation (in the subgroup of patients with premature discontinuation).
- The following will also be presented:

- Frequency and percentage of HHD patients included before and after removal of the matching according to the initial protocol as of 13/10/2020
- Frequency and percentage of HCC patients included before and after removal of matching according to the initial protocol dated 13/10/2020
- Frequency and percentage of HCC patients matched to HHD patients

6.2. Definition of Populations of Analysis

The following populations of analysis are defined:

- Patient population
- Exposed-unexposed population
- Vigilance population
- Centre population

6.2.1 Patient population

The patient population consists of all patients included in the study for whom:

1. *At least the CRF is available and at least one item has been completed;*
2. *The "exposed" or "unexposed" category has been determined;*
3. *The inclusion and exclusion criteria have been met.*
4. *No major deviations from the protocol*

6.2.2 Exposed-unexposed population

The "exposed-unexposed" (EUE) *population* is a subpopulation of the patient population. It *consists of all exposed* and matched unexposed patients (matching according to the initial protocol or matching after randomisation among haemodialysis patients in the centre) and *without major deviation from the protocol*.

The two groups are defined as follows:

a. HHD patient: This subgroup comprises all patients undergoing daily home haemodialysis (HHD).
b. HCC patients: This subgroup comprises all patients receiving conventional centre-based haemodialysis (HCC).

6.2.3 Vigilance Population

The vigilance population is defined as all patients included in the study who underwent at least one haemodialysis session during the study.

6.2.4 Active centre population

The active centre population consists of all centres that have included at least one patient in the study.

In the rest of the SAP, the terms 'sites' and 'centres' are used interchangeably.

6.3. Treatment Assignment Errors

This section is not applicable.

6.4. Other Analysis Population

An additional population is defined: The centre population consists of all centres where the study was implemented.

6.5. Major Protocol Deviations

Major protocol deviations are defined as the occurrence of at least one of the following criteria:

- Any violation of at least one of the inclusion or exclusion criteria. A violation corresponds to at least one of the following conditions:
    - Inclusion criterion marked "No" or not marked.
    - Age < 18 years: The criterion "age ≥ 18 years" will be verified based on the age entered on the eCRF:

      [DemoAge] <18
    - Unexposed subject ≥ 80 years old. Unexposed subjects must also be under 80 years of age, and this criterion will be verified based on the age entered on the eCRF:

      [DemoAge] > 80
    - Non-inclusion criterion ticked "yes" or not ticked.
- Other major violation criteria defined prior to database lock and during the data review meeting for final analysis with the sponsor, experts and statistician.

6.6. Definition of Analysis Subgroups

The following subgroups are defined for the analysis:

1. Wristband subgroup: This subgroup consists of all subjects from the exposed and unexposed populations with at least one data recorded by the electronic wristband. This subgroup is only defined for the final analysis.
2. RLS subgroup: This subgroup consists of all patients with restless legs syndrome (RLS), i.e. RLS checked as "Yes" at inclusion ([ComPrescSDJSR]=1) and/or at least one of the follow-up visits (V1 to V12) ([DonClinSDJSR]=1).
3. Lockdown subgroup: All subjects in the patient population included before 1st February 2020 and with at least one data recorded by the connected wristband during and before or after the lockdown period in France linked to the COVID-19 epidemic (25 March to 11 May 2020) or with a Prochaska DiClemente stage or at least one data available on physical activity from the "lockdown" questionnaire.

7. VARIABLES OF ANALYSIS

7.1. Characteristics of the sites

The data collected on the "centre questionnaire" are:

- Centre department - Categorical variable
- Type of centre - Categorical variable (comprehensive haemodialysis centre /UDM/Self-dialysis)
- Number of patients receiving HDD – continuous variable;
- Year of 1st HHD prescription - ordinal categorical variable.

7.2. Demographic characteristics and inclusion data

Demographic and inclusion data are defined by the following parameters, presented with the type and unit where applicable:

7.2.1 Socio-demographic data

- Age (year) – continuous variable;
- Gender – dichotomous variable (Female/Male);
- Height (centimetres (cm)) - continuous variable;
- Weight (kilogrammes (kg)) - continuous variables:
  - Before dialysis
  - After dialysis
  - Consultation
- Body mass index (BMI) (kg/m²) – continuous variable (2 decimal places); Derived parameter = weight (kg)/height^2(m). The weight after dialysis will be used to calculate the BMI if the weight at the consultation is missing.

  Body mass index: Ordinal categorical variable, derived parameter. Body mass index categories according to the WHO classification will be derived from BMI[3] :

  a. Underweight if BMI <18.50 kg/m²;

  b. Normal weight if BMI is in the range [18.50; 25.00[;

  c. Overweight if BMI is in the range [25.00; 30.00[;

  d. Obese if BMI ≥ 30.00 kg/m².
- Smoking status – categorical variable (non-smoker/smoker/former smoker);
- Employment status – categorical variable (employed: full-time/part-time, unemployed, retired, other);
- Transportation option to the centre – categorical variable (private car, licensed taxi, ambulance, light medical vehicle, other);
- Distance from home to centre (kilometres (km)) – continuous variable;

7.2.2 Medical history

The characteristics of the pathology and the patients' medical history are defined by the following parameters, presented with the type and unit where applicable:

1. Characteristics of the pathology:
   - Pathology responsible for chronic renal failure (CRF) – categorical variable – derived parameter (MedDRA coding);
   - The time (years) between inclusion and diagnosis of CRF will be calculated as follows: (Date of inclusion – Date of diagnosis) /365.5 - categorical variable. When the date of diagnosis only includes the year, the time will be calculated between the year of the date of inclusion and the year of diagnosis;
   - Length of haemodialysis (months) will be calculated as follows: (Date of inclusion – Date of initiation of haemodialysis) /30.5 - Quantitative variable. When the date of initiation of haemodialysis is incomplete, the delay will be calculated as follows (Year of inclusion – Year of initiation of haemodialysis) * 12;
   - Previous transplant(s) – binary variable;
   - Transplant status (registered, assessment in progress, not transplantable) – categorical variable;

2. Dialysis characteristics (see §7.4.2).

7.2.3 Comorbidities

The following specific comorbidities (binary variables) are recorded in the CRF: diabetes, arterial hypertension, heart failure, arrhythmia, asthma, chronic bronchopathy, cardiovascular complications.

7.2.4 Concomitant treatments

All treatments will be coded using the ATC code, version in force at the start of the coding process, available at https://www.whocc.no/atc_ddd_index/[4] .

The treatment and therapy data for patients at inclusion are defined by the current drug treatments (name of treatment, dose, unit, route and frequency) which are collected on the "Treatments" form in the CRF. The treatment categories are as follows, with the corresponding ATC code and category[4] :

- Iron: B03A - Iron preparations
- Erythropoietin (EPO): B03XA01 - Erythropoietin
- Antihypertensives: C02 - Antihypertensives
- Phosphate binders: V03AE02 - Sevelamer
- Vitamin D – 4th level: A11CC - Vitamin D and analogues
- Calcium supplements:
  - Without vitamin D: A12A – Calcium
  - With vitamin D: A12AX - Calcium, combinations with vitamin D and/or other drugs
  - Calcium carbonates: A02AC01, A12AA04 - Calcium Carbonate
  - Calcimimetics: H05BX01 – Cinacalcet
- Bicarbonates:
  - A02AH – Antacids with sodium bicarbonate
  - B05CB04, B05XA02 - Sodium Bicarbonate
- Potassium chelator:
  - V03AE01 - Polystyrene sulphonate
  - V03AE09 - Patiromer Calcium
- Oral anticoagulants:
  - B01AA - Vitamin K antagonists
  - B01AF01 – Rivaroxaban
  - B01AF02 – Apixaban
  - B01AE07 - Dabigatran Etexilate
- Heparin (other than for dialysis session):
  - B01AB - Heparin group
  - B01AC - Platelet aggregation inhibitors excl. Heparin
  - C05BA - Heparins or heparinoids for topical use
  - B01AB01, C05BA03, S01XA14 - Heparin
  - B01AB51, C05BA53 - Heparin, combinations
- Diabetes treatments: A10 - Drugs used in Diabetes
- Painkillers: N02 - Analgesics
- Medical treatments for RLS:
  - N04BC04 - Ropinirole
  - N04BC05 - Pramipexole
  - N04BC09 - Rotigotine
- Medical treatments for sleep disorders (anxiolytics, muscle relaxants, hypnotics):
  - N05B – Anxiolytics
  - N05C - Hypnotics and sedatives

- M03B - Muscle relaxants, centrally acting agents
- M03C - Muscle relaxants, directly acting agents

7.2.5 Clinical examinations and laboratory data

Clinical examination and laboratory data collected from patients at enrolment and at each monthly and quarterly visit are defined by the following parameters:

1. Relevant biological data (latest available blood sample): *haemoglobin, glycated haemoglobin, calcium, phosphorus, PTH, bicarbonate, alkaline phosphatase, ferritin, transferrin saturation coefficient, albumin, sodium, pre-dialysis β2-microglobulin level*.
2. Urinalysis with collection method (sample, 12-hour or 24-hour urine collection): creatininuria, urea, proteinuria, microalbuminuria. Derived data recalculated over 24 hours will be used for analysis; the results of a 12-hour urine collection will be multiplied by 2.

7.3. Main Criterion

The primary endpoint is the total average number of steps taken per day at 3 months, 6 months, 9 months and 12 months. This continuous parameter is derived from data collected by the connected wristband. It is defined as follows:

$$Mean\ number\ of\ steps\ during\ the\ period = \frac{Sum\ of\ the\ number\ of\ steps\ over\ the\ period}{Number\ of\ days\ the\ wristband\ was\ worn\ during\ the\ period}$$

The wristband was to be worn for 7 days (see §8.5.2). The wristband data considered for analysis will be the number of steps recorded during the week preceding days 90, 180, 270 and 365, corresponding to 3, 6, 9 and 12 months respectively. If unavailable, data from any week of at least 5 consecutive days within ±42 days of these time points will be used.

A valid wristband-wear day is defined as any day during which the wristband is worn and records at least 100 steps. Because the device does not provide the actual daily wear time, wear duration will not be used in estimating the number of steps per day. However, the total wear duration over the study period will be calculated as the sum of the minutes classified by the device as sedentary, low-activity, moderate-activity, and high-activity.

If the wristband is worn for fewer than 5 consecutive days, the cumulative data from any 7-day wear period within the corresponding time window will be used. Conversely, if the wristband is worn for fewer than 7 days during the relevant window, the cumulative data available for that entire period will be considered.

If the wristband is worn continuously, the data from the week closest to days 90, 180, 270, and 365 will be used.

This amendment introduces an additional derived variable: the average number of steps during the wear period, calculated as the total number of steps recorded over the period divided by the total wear time during that period.

7.4. Secondary Criteria

7.4.1 Physical activity

The secondary criteria for the study are:

1. *Connected wristband:*
    - *Wearing of the wristband since the last consultation;*
    - *Synchronisation completed.*

2. *The average number (Nbr) of steps per day by HCC patients on days without dialysis and days with dialysis* will be calculated based on all data available while patients wore the wristband, as follows:

$$Mean\ number\ of\ steps\ on\ days\ of\ dialysis = \frac{Sum\ of\ the\ number\ of\ steps\ on\ the\ days\ of\ dialysis}{Number\ of\ days\ of\ dialysis\ the\ wristband\ was\ worn}$$

$$Mean\ number\ of\ steps\ on\ nondialysis\ days = \frac{Sum\ of\ the\ number\ of\ steps\ on\ nondialysis\ days}{Number\ of\ nondialysis\ days\ the\ wristband\ was\ worn}$$

Dialysis and non-dialysis days will be identified from the patient diary. For each analysis period, all observations for which diary dates and wristband wear dates overlap will be retained, provided that the period includes at least one dialysis day and at least one non-dialysis day. Only data from overlapping dates will be used in the analyses.

Delta of the average number of daily steps with and without dialysis: Average number of steps on dialysis days - Average number of steps on non-dialysis days. This continuous derivative parameter will be estimated at each period.

3. Activity times per day will be considered for analysis as for the primary criterion at 3, 6, 9 and 12 months. Activity time is collected via the connected wristband and is defined as follows for each parameter:

$$Mean\ duration\ in\ minutes\ over\ the\ period = \frac{Sum\ of\ minutes\ during\ the\ period}{Number\ of\ days\ the\ wristband\ was\ worn\ during\ the\ period}$$

The activity time parameters collected daily by the wristband are:

- Sedentary minutes
- Minutes of low activity
- Moderately active minutes
- Very active minutes

The average daily time (in minutes) spent in each activity level will be calculated separately for days without dialysis and days with dialysis:
E.g.: Sedentary time in the period considered

$$\text{Mean sedentary time on nondialysis days over the period, in minutes} = \frac{\text{Sum of sedentary minutes on nondialysis days during the period}}{\text{Number of days the wristband was worn on nondialysis days during the period}}$$

$$\text{Mean sedentary time on dialysis days over the period, in minutes} = \frac{\text{Sum of sedentary minutes on dialysis days during the period}}{\text{Number of days the wristband was worn on dialysis days during the period}}$$

4. Average quarterly physical activity

This secondary criterion is added for the final statistical analysis.

All available data from the wristband will be taken into account per quarter: [0-90 days], [91-180 days], [181-270 days], [271-365 days].

$$\text{Mean quaterly steps} = \frac{\Sigma \text{ steps during the quarter}}{\text{Number of days the wristband was worn during the quarter}}$$

5. *Patient diary data*

A double-sided page was sent to doctors at the end of January 2020 to supplement the patient diary (which previously consisted of only one page). These two pages, which are provided with the patient diary, were to be completed by the patient and enable additional information to be collected on physical activity, upon inclusion in the study and at withdrawal from the study:

- Physical activity:
  Subjects will be categorised at study inclusion and exclusion according to Prochaska and DiClemente's 5 stages (stages of behaviour change)[5] for physical activity. The Prochaska and DiClemente stage is derived from the 4 questions below, as follows:

1. I do not currently engage in physical activity	*No*	*Yes*
2. I intend to engage in physical activity in the next 6 months	*No*	*Yes*
3. I exercise regularly<br>*Regularly = 3 times a week or more for 20 minutes*	*No*	*Yes*
4. I have been exercising regularly for more than 6 months<br>*Regularly = 3 times a week or more for 20 minutes*	*No*	*Yes*
The stages of physical exercise are defined as follows:<br>• If the answer to question 1 = Yes and question 2 = No, then Stage = Precontemplation<br>• If the answer to question 1 = Yes and question 2 = Yes, then stage = Contemplation<br>• If the answer to question 1 = No and question 3 = No, then stage = Preparation<br>• If the answer to question 3 = Yes and question 4 = No, then stage = Action<br>• If the answer to question 3 = Yes and question 4 = Yes, then stage = Maintenance		

- Details of physical activity at inclusion and at end of study, as well as the number of times per week and total duration (in minutes) of each type of activity and total duration:
  1. Running – Binary (Yes/No)
  2. Cycling – Binary (Yes/No)
  3. Walking – Binary (Yes/No)
  4. Brisk walking – Binary (Yes/No)
  5. Gardening – Binary (Yes/No)

  In addition, the number of activities performed per week will also be calculated by adding up the number of yes answers for the above activities.
- Patient assessment of their activity at the end of the study compared to inclusion in the study category (increased/the same or almost the same/decreased)
- The change in physical activity between inclusion and the end of the study is defined as follows:

$$\Delta \ aphysical\ activity\ (minutes) =$$
$$\sum total\ duration\ of\ physical\ activity\ at\ inclusion\ (min) - \sum \begin{matrix} total\ duration\ of\ physical\ activity \\ at\ end\ of\ study (min) \end{matrix}$$

7.4.2 Dialysis characteristics

1. *The characteristics of HHD patients* are defined by all the parameters of the inclusion visit detailed in sections 7.2 ,7.2.2 ,7.2.3 and 7.2.5 above.
2. Dialysis characteristics collected at inclusion and at each monthly and quarterly visit:
   - Dialysis location (at enrolment and at the end of the consultation) - Categorical variable;

- Prescription at the end of the consultation:
  i. Type of centre – Categorical variable;
  ii. Number of weekly sessions - Categorical variable (1 session, 2 sessions, etc.);
  iii. Dialysis duration (prescription) (hours) - Derived quantitative variable: Number of hours*60 + number of minutes;
  iv. Type of vascular access (arteriovenous fistula, catheter) - Categorical variable;
  v. Type of needle (blunt or sharp) – Categorical variable;
  vi. Type of puncture (ButtonHole or standard puncture) - Categorical variable;
  vii. Needle size (G) - Categorical variable;
  viii. Anticoagulation (IU) - Categorical variable, conversion from mg to IU as follows:
     Low molecular weight heparin (LMWH) (IU)[6] : 1 IU = 100 mg
  ix. Blood flow rate (mL/min) - Quantitative variable;
  x. Dialysate composition (Ca2+, K+, Lactate, HCO3, Citrate) – Binary variables and quantitative variable in mmol/L for each element in the dialysate composition.
- Data collected only from HCC patients (inclusion and every quarter):
  - Dialysis method (HD, pre-dilution HDF, post-dilution HDF) - Categorical variable;
  - Total convective volume per session for HDF patients (L/session) – Quantitative variable;
  - Plasma volume control technique (yes/no) – Binary variable
- Data collected only from HHD patients:
  - Variable derived at inclusion: Time between start of training and inclusion in days = Training start date [CarDialDomFormaDte] – date of inclusion consultation [VisDte]+1
  - Prescribed convective volume per session (L), Quantitative variable collected at inclusion and at quarterly visits;
  - Dialysate flow rate (mL/min), quantitative variable collected at inclusion and at quarterly visits;
  - Weekly number of sessions completed, Ordinal variable,
  - Usual duration of dialysis, quantitative variable.

3. Reason for discontinuation at 12 months and reasons for discontinuation, haemodialysis discontinuations are collected on the "Discontinuation of haemodialysis or daily dialysis" form
   HHD discontinuation is defined by one of the following reasons:
   - Impact on family life
   - Deterioration in quality of life
   - Feeling of loneliness
   - Lack of follow-up
   - Deterioration in health that no longer allows treatment at home

   The time to HHD discontinuation, in days, is defined as follows:

   Date of last HHD session – Date of inclusion + 1 day

7.4.3 Clinical data

Clinical data are collected at each visit (inclusion, monthly and quarterly) and are continuous variables unless otherwise specified.

7.4.3.1. Weight change

The weight data used for the analysis are those collected in the CRF and the corresponding derived parameters (continuous).

Pre- and post-dialysis weight from the last dialysis session before the visit + consultation weight. In addition, the difference in weight between post- and pre-dialysis weight will be calculated in grams as follows:

Δ Dialysis weight (in grams) = [post-dialysis weight (kg) - pre-dialysis weight (kg)] *1000

The parameters used to analyse changes in patient weight in the study are as follows:

- Patients' weight before dialysis at each follow-up visit (grams (g))
- Patients' weight after dialysis at each follow-up visit (g)
- Weight lost during dialysis in grams (g) = [Post-dialysis weight (kg) – Pre-dialysis weight (kg)]*1000
- Δ Weight at consultation between the 1st and last visit in the study:

Δ Weight at visit (g) = [weight at last visit (kg) - weight at visit no. 1 (kg)] *1000

7.4.3.2. Change in blood pressure

The data analysed will be the data collected on the CRF and the corresponding derived parameters (continuous). Blood pressure before/after dialysis (collection of the last 3 available measurements, on 3 different days) is collected at inclusion and at each visit. The average of the 3 measurements will be calculated independently for systolic blood pressure (SBP) and diastolic blood pressure (DBP), before and, independently, after dialysis (0 decimal places, rounded to the nearest integer), as follows:

$$Mean\ BP\ (mmHg) = \frac{\Sigma documented\ BP}{Number\ of\ documented\ BP}$$

The delta between the average of the 3 measurements before and the average after dialysis will also be calculated at inclusion and at each visit as follows:

Δ BP (mmHg) = Average of the 3 measurements after dialysis - Average of the 3 measurements before dialysis

These parameters will be calculated independently for SBP and DBP.

7.4.4 Hospitalisations and other complications

1. *Hospitalisations, duration and departments:* these are defined by the following data collected at each monthly visit:
   - Number of hospitalisations between 2 visits – Ordinal categorical variable from 0 to 3;
     For each of the 3 possible hospitalisations: Reasons, Hospitalisation department, Duration (days).
   - The following derived parameters will be calculated for each patient over the entire follow-up period of the study:
     - Total number of hospitalisations during the study – Quantitative variable;
     - Total length of hospitalisation (days) during the study - Quantitative variable;
     - Percentage of hospitalisation duration during the study: total hospitalisation duration in days* 100 / total follow-up duration in days in the study – Quantitative variable (1 decimal place)
   - As the length of hospitalisation depends on the total duration in the study, a ratio will also be calculated as follows: $\frac{\text{total length of hospitalisation (days)}}{\text{total duration in the study (days)}} \times 100$
     The total duration of participation in the study is defined as the difference in days between the date of inclusion and the date of study completion for the patient + 1.

2. *Cardiovascular complications* collected via the adverse event form will be coded using the MedDRA dictionary, version 24.1 or later, in effect at the start of the coding process. All cardiovascular complications correspond to the following *System Organ Classes* (SOC):
   - "Cardiac disorders -10007541" with the exception of complications corresponding to the following *High Level Group Term* (HLGT):
     - "Endocardial disorders - 10014662"
     - "Cardiac Tumours - 10007590"
   - "Vascular disorders - 10047065" with the exception of complications corresponding to the following HLGT:
     - "Vascular infections and inflammations - 10082207"
     - "Vascular lesions - 10047079"
     - "Varicose veins - 10057165"
     - "Lymphatic vessel disorders - 10025231"

   The time to onset of a cardiovascular complication will be calculated as follows, in days:

   Time to cardiovascular complication = Date of occurrence of the adverse event – Date of inclusion + 1

3. Surgical intervention: PMSI code. As PMSI codes were rarely completed in the CRFs, this criterion will not be analysed.

7.4.5 Self-administered questionnaires

1. *Kidney Disease Quality of Life Instrument (KDQOL-SF™) questionnaire:* the calculation of the score is detailed in the reference Hays, R.D., et. al[7] *;*
2. *The average sleep duration* (in hours) will be calculated as follows using data on the number of minutes of sleep recorded by the wristbands on the days when the wristband was actually worn (the number of days the wristband was worn as defined in section 8.5.2).

$$Mean\ sleep\ duration\ (\text{hours}) = \frac{\Sigma minutes\ of\ sleep\ during\ the \frac{period}{60}}{\text{Number of days the wristband was worn during the period}}$$

For a given day, the sleep duration will add up the minutes of sleep recorded between 8:00 p.m. (date of the given day) and 12:00 p.m. the following day.
The following two criteria are added for statistical analysis: wakefulness duration and number of awakenings, data from the wristband, which will be transformed as above to calculate the average wakefulness duration/day (in hours) and the average number of awakenings/day.

3. *The percentage of patients with RLS* is defined by the number of subjects who answered yes to the item
   IRLS (International Restless Legs Syndrome Rating Scale) questionnaire: the severity of RLS symptoms is categorised using the IRLS questionnaire by adding up the points from the questionnaire responses[8] :
   - Mild syndrome: ≤10 points.
   - Moderate syndrome: 11 to 20 points.
   - Severe syndrome: 21 to 30 points.
   - Very severe syndrome: 31 to 40 points.

An additional derived criterion will be defined into three categories: SJRS at the end of the study DonC linSDJSR last consultation in the study = 1 / presence of at least one SJRS during the study and no longer present at the end of the study [ComPrescSDJSR = 1 or at least DonC linSDJSR = 1] and DonC linSDJSR last consultation in the study = 0 / no SJRS during the study sum (ComPrescSDJSR, DonC linSDJSR) = 0. The severity of RLS does not enter into the definition of this derived criterion.

4. *PSQI (Pittsburgh Sleep Quality Index) questionnaire:* Following the sponsor's decision, the PSQI questionnaire will not be analysed.

7.5. Vigilance parameters

7.5.1 Adverse events

Adverse events are recorded on the adverse event (AE) form. All AEs will be coded using the MedDRA dictionary, version 24.1 or any later version in effect at the start of the coding process.

Where applicable, the number of deaths will be presented in each exposure group.

For each AE/serious adverse event (SAE), the duration of the AE/SAE will be calculated in days (continuous parameter).

The frequency of AEs per patient will also be calculated.

In the exposed group, for each AE/SAE, the time interval between the date of the first HHD session and the date of occurrence of the AE/SAE will be calculated in days (continuous parameter).

7.5.2 Vital signs and physical examinations

Vital signs and clinical examination data are included in the secondary efficacy criteria.

7.5.3 Laboratory parameters

Biochemical data are collected at inclusion and at each visit and are efficacy parameters.

7.6. Other parameters

7.6.1 Physical activity and confinement

In the context of the COVID-19 health crisis, Prochaska and DiClemente's stage and patients' physical activity during the first lockdown period from 25 March to 11 May 2020 and after the lockdown ended were to be collected on a specific form entitled "Lockdown period – Physical activity questionnaire" (see §7.6.1), corresponding to amendment no. 3 to the protocol.

For the wristband data, the period after lockdown is defined as the interval [1st June 2020 to 31 July 2020].

The following parameters will be derived:

- Total number of days the wristband is worn during lockdown and total number of days the wristband is worn after lockdown. All wristband data available for each period will be used (see §7.3 for the definition of a day the wristband is worn).
- The average number of steps/day during the lockdown period will be calculated using all the data available from the wristband during the lockdown period.
- The average number of steps/day at the end of lockdown will be estimated from all the data available from the wristband for the months of June and July 2020 (the period considered to be the end of lockdown).
- Delta of the average number of steps/day = average number of steps/day during the lockdown period - average number of steps/day at the end of lockdown.
- The average daily time for each activity will be calculated using all the data available from the wristband during the period of interest (lockdown/end of lockdown).
- Delta of sedentary time (minutes): average sedentary time/day during lockdown - average sedentary time/day after lockdown.

- Prochaska and DiClemente stage calculated independently for the period during lockdown and after lockdown (lockdown log data).
- Details of physical activity during and after lockdown, as well as the number of times per week for each type of activity listed above during and after lockdown, total duration of all activities (minutes) and delta of total duration (total duration during lockdown – total duration after lockdown). The calculation will be made independently for each period: during and after lockdown (lockdown log data).

The Prochaska and DiClemente stage will be estimated using the method described in section 7.4.1.

7.6.2 Characteristics of follow-up visits

- Number and percentage of patients who attended the visit
- Time between inclusion visit and visit (weeks): (date of visit – date of inclusion in the study +1) /7
- Patient status: continuing haemodialysis at the centre/continuing haemodialysis at home/stopping haemodialysis at home and returning to the centre/stopping haemodialysis completely

7.6.3 Quarterly general information data (V3, V6, V9 and V12):

The data (see 8.2.1) are collected quarterly:

- *Employment status;*
- *Transportation option to the centre;*
- *Distance between home and centre*.

7.6.4 Premature withdrawal from the study

- Number and percentage of subjects who withdrew prematurely (denominator = patient population and other denominator exposed population – unexposed population)
- Total duration of participation in the study in weeks will be calculated as follows:

  (Date of last visit or last telephone contact – Date of inclusion +1) /7
- Reasons for study withdrawal in the event of premature withdrawal: death, withdrawal of consent, kidney transplant, lost to follow-up, other.

The following time periods are considered:

- Transplant time (days) = (Transplant date – inclusion date) +1
- Time to death (days) = (date of death – date of inclusion) +1
- Total duration of participation in the study (days) = (Date of last contact – Date of inclusion) +1

7.7. Covariates

The adjustment variables in the protocol are as follows:

- *Age* (year) - Continuous variable.
- *Gender* - Binary variable
- *Duration of haemodialysis at inclusion* (months) - Continuous variable

- *BMI at inclusion* (kg/m²) - Continuous variable or categorical if BMI distribution does not follow a normal distribution.
- *Presence or absence of heart failure at inclusion* - Binary variable

There is a strong correlation between heart failure, diabetes and high blood pressure, for this reason the following parameters are removed from the adjustment variables

- *Presence or absence of diabetes mellitus at inclusion* - Binary variable
- *Presence or absence of hypertension at inclusion* - Binary variable

Furthermore, in the event of model convergence issues, only one of the two parameters, age or length of dialysis, will be included in the model, in addition to the other adjustment variables.

8. STATISTICAL ANALYSES

8.1. General

Statistical analysis will be performed using SAS software version 9.4 and SAS/STAT 15.3 or later (SAS Institute, North Carolina, USA).

The parameters will be described classically:

- Continuous variables (e.g. age, weight, height, BP) will be described by the frequency of documented data, frequency of missing values, the mean, standard deviation, median, quartiles (Q1, Q3) and extreme values.
- Categorical variables (e.g. gender) will be described by frequency and percentage of each documented modality. The frequency of missing data will also be presented.

All results will be presented blinded to *the centre* and the patient*.*

Statistical tests will be two-tailed with a statistical significance threshold of 5%. The normality of continuous biological parameters will be verified and the hypothesis of normal distribution rejected if at least two tests are statistically significant at the 5% threshold. A visual check will also be performed. If the assumption of normality of a distribution is rejected, an attempt will be made to normalise the distribution by a simple transformation. If no simple transformation can normalise the variable, then tertiles will be used in the statistical analysis.

Unless otherwise specified, all data from the questionnaires will be described by exposure group (HHD and HCC) independently in the patient population and in the exposed-unexposed population.

The analyses will be performed in the patient population and the exposed-unexposed population if the latter represents less than 85% of the patient population.

For the exposed-unexposed population: If the percentage of matched subjects is greater than 90%, the comparison between the two groups at a given time during the study will be performed using a conditional logistic regression model to take into account the matching of exposed and unexposed cases; otherwise, *the two groups will be compared in the same way as in the patient population.*

If the percentage of matched subjects is less than 80%, then the comparison between the two exposure groups will be performed using a generalised linear mixed effects model with the centre as a random effect.

Unless otherwise specified, a generalised linear mixed model will be used with:

- *The specific intercept for each patient and the slope of the measurement times as random effects*
- *The other parameters tested as fixed effects.*

The model will be tested with three different variance-covariance matrix structures: first-order heterogeneous autoregressive (ARH (1)), Toeplitz, and no structure. If there is a convergence problem with the model using the three proposed matrix structures, another covariance structure may be tested, namely an unstructured matrix parameterised using Cholesky factorisation. The Akaike information

criterion (AIC) will be used to choose the most appropriate covariance structure for repeated measures for a given model.

The analysis will be performed with adjustment for the parameters listed in section 7.7. *Level 2 interactions will be tested at the 5% threshold, and only interactions that are significant at the 5% threshold will be retained in the model*. The analyses that will be adjusted are specified in the present document.

For ordinal categorical parameters, a cumulative logit function will be used if the assumption of proportional odds is not rejected.

8.2. Hypotheses to be tested

H0: Number of steps taken by subjects in HHD = Number of steps taken by subjects in HCC, *i.e.* OR=1

H1: Number of subjects in HHD ≠ Number of subjects in HCC, *i.e.* OR≠1

8.3. Grouping of Sites

Data from all centres will be grouped together for the analysis. The centre effect will not be taken into account as subjects are already matched by centre at inclusion.

8.4. Interim Analyses

In the study protocol version 9.0 dated 10/05/2021, an interim analysis is planned. However, due to a delay in the study schedule caused by the SARS-CoV-2 epidemic, the study sponsor has decided to cancel this interim analysis. Only the final analysis will be performed.

8.5. Time Points for Analysis

8.5.1 Time point-visit

Data of follow-up visits will be included in the analysis if the follow-up date falls within the following intervals:

Monthly visits:

- 1 month: [Inclusion date +15 days; Inclusion date +45 days]
- 2 months:] Date of inclusion +45 days; Date of inclusion +75 days]
- 4 months:] Inclusion date +105 days; Inclusion date +135 days]
- 5 months:] Inclusion date +135 days; Inclusion date +165] days
- 7 months:] Date of inclusion +195 days; Date of inclusion +225 days]
- 8 months:] Date of inclusion +225 days; Date of inclusion +255 days]
- 10 months:] Date of inclusion +285 days; Date of inclusion +315 days]
- 11 months:] Date of inclusion +315 days; Date of inclusion +345 days]

Quarterly visits:

- 3 months:] Date of inclusion +75 days; Date of inclusion +105 days]
- 6 months:] Date of inclusion +165 days; Date of inclusion +195 days]
- 9 months:] Date of inclusion +255 days; Date of inclusion +285 days]
- 12 months:] Date of inclusion +345 days; Date of inclusion +405 days]

If two visits are available during the same period, the visit closest to the theoretical date (1, 2, 3 months, etc.) will be considered.

8.5.2 Time point for wristband

The data from the wristband will be taken into account as follows:

- First 7 days of each calendar month
- If the number of days of data available during the first week of a month is less than 7 days, then the data from the first 7 days of the following month will be considered.
- See §7.3 for cases where neither of the above two possibilities applies.

8.5.3 Calculation of time limits and censoring

For survival models, unless otherwise specified, subjects who did not experience the event during the study will be censored at 365 days or on the date of last contact, date of death, date of kidney transplant, whichever occurred first.

8.6. Method for replacing missing data

For the primary endpoint, as quarterly data are included in the analysis in addition to the inclusion data, a missing quarterly value will be replaced by the last available value for that quarter. If no data are available for the quarter, the missing value for the quarter in question will not be replaced.

For data on employment status, mode of transport and distance from home to centre, any missing data will be replaced as follows: For the last visit of the study, if the data from the two previous visits are identical, the corresponding missing data will be replaced by the latter. For an intermediate quarterly visit, if the data between the previous visit and that of the visit following the visit with missing data are the same, then the missing data will be replaced by the data from the previous visit.

No replacement will be made for other missing data, with the exception of incomplete dates, which may be completed to allow the calculation of intervals as follows, provided that the replacement does not result in a negative time period:

- For incomplete dates where the day is missing, the day will be replaced by 15.
- Except for visit dates, other incomplete dates where the month is missing will have the month replaced by June (06).

8.7. Characteristics of the sites

The total number of centres and the number and percentage of active centres will be presented.

The characteristics of active centres and data from the questionnaire "Questionnaire on the efficiency of daily home haemodialysis compared to conventional haemodialysis in centres 3 times a week" will be described in the population of active centres.

The total number of patients included (categorical parameter) per site will also be presented in the population of active centres.

9. EVALUATION OF DEMOGRAPHIC AND INCLUSION CHARACTERISTICS

Socio-demographic and inclusion data will be described by exposure group independently in the patient population and *in the exposed-unexposed population*.

For *demographic and inclusion* characteristics*, the two subgroups will be compared using a chi-square test (or Fisher's non-parametric test if the theoretical sample size is < 5) for categorical parameters and a t-test (or non-parametric test if the assumption of normal distribution is rejected) for continuous quantitative parameters.* However, if matching can be considered (see §8.1), then univariate analyses will be performed, using a conditional logistic regression model, to compare the two groups.

9.1. Demographic data

Socio-demographic data will be described by exposure group.

9.2. Medical History

The characteristics of chronic kidney disease (CKD) will be described by exposure group.

Comorbidities will be described by exposure group independently in the patient population and in the exposed and unexposed populations.

The frequencies of pathologies responsible for ESKD and comorbidities will be compared between exposed and unexposed patients using a chi-square test on the exposed and unexposed populations. The time between inclusion and initiation of haemodialysis will be compared using a t-test on the same population.

9.1. Concomitant treatments (inclusion and follow-up)

Concomitant treatments will be presented at inclusion and at each visit by category as defined in section 7.2.4. The frequency and percentage of each category will be presented by exposure group independently in the patient population and in the exposed and unexposed populations.

In addition, a listing will also be edited with the following information:

- Patient number
- Name of medication
- The 5 levels of ATC coding
- Start and end dates
- In progress
- Reason for prescription
- Dosage, unit, route and frequency

9.2. Clinical examinations and laboratory data

Blood and urinary data will be described at inclusion along with follow-up visit data by exposure group.

9.3. Dialysis characteristics

Dialysis characteristics and prescriptions at the end of the inclusion consultation will be presented by exposure group independently in the patient population and in the exposed and unexposed populations. The analysis will be descriptive only.

10. ANALYSIS OF EVALUATION CRITERIA

10.1. Analysis of the Main Criterion

The primary criterion is the average total number of steps per day. The average will be calculated based on the days on which the wristband was worn during the 7 days per month specified in the study.

The average total number of steps will be analysed using a generalised linear mixed model with adjustment for the parameters listed in section 7.7 to compare the exposed and unexposed populations.

If the number of steps does not follow a normal distribution, a simple transformation will be attempted to normalise the variable. If the variable cannot be normalised simply, it will be categorised into defined quartiles, across the entire population, based on the number of steps at inclusion. An ordinal mixed model will then be used, following the methods and adjustments described in section 8.1 .

The number of steps will also be described by quarter as defined in the section 7.3, in each exposure group in the patient population and the exposed-unexposed population.

The primary endpoint analysis will only be performed during the final data analysis.

10.2. Analysis of Secondary Endpoints

10.2.1 Physical activity

1. Data from the connected wristband will be described in each exposure group in the connected wristband subgroup.
   The number of subjects who wore the wristband during the following periods will also be presented:
   - During the study
   - Before the first lockdown
   - During lockdown
   - After lockdown
2. The average number of steps per day in HCC patients on non-dialysis days and on dialysis days, and the delta in the number of steps, will be described using standard descriptive statistics in the HCC subgroup.

   If the number of steps follows a normal distribution, a comparison between days with and without dialysis will be performed using a paired t-test or the non-parametric equivalent of the signs test if the average number of daily steps does not follow a normal distribution. This comparison will only be made in the HCC patient subgroup.
3. Physical activity times will be described in each exposure group and independently in the patient population and the exposed-unexposed population. The change in average daily sedentary time will be analysed using a generalised linear mixed model adjusted for the parameters listed in paragraph 7.7 .
4. Data from the patient diary (inclusion and end of study) will be described according to the Prochaska and DiClemente stages in each exposure group and independently in the patient population and the exposed-unexposed population.
   In addition, the Prochaska and DiClemente stage at inclusion will be cross-referenced with that at the end of the study.

10.2.2 Dialysis characteristics

1. The characteristics of HD patients will be described in the exposed subgroup and independently in the patient population and the ENE population.
2. *The haemodialysis modalities of patients on HHD* will be described independently in HHD subjects included in the patient population and independently in the exposed-unexposed population.
3. The haemodialysis modalities for HCC patients will be described independently for those included in the patient population and those included in the exposed-unexposed population.
4. Other dialysis characteristics, collected at inclusion and at each monthly and quarterly visit, will be described in each exposure group and independently in the patient population and the exposed-unexposed population.
5. The frequency and percentage of premature discontinuation and reasons for discontinuation will be described in the HHD group.
   The HHD discontinuation rate at 12 months (52 weeks) *will be estimated in HHD subjects using the Kaplan Meier method*. Transplanted or deceased subjects will be censored on the date of transplantation/death.
6. *The number and percentage of subjects who underwent transplantation during the study period will be presented in the two* exposure *groups* of the patient population. The rate of subjects

who underwent transplantation at 12 months (52 weeks) will be estimated using the Kaplan-Meier method, independently in each of the exposure groups in the patient population. The rate will also be estimated in each exposure group using a competing risk model with death as the competing risk.

10.2.3 Clinical data

10.2.3.1. Weight change

Pre- and post-dialysis weight and Δ Dialysis weight will be described at each visit by exposure group independently in the patient population and the exposed-unexposed population.

The change in patient weight will be analysed using a linear regression model for repeated data with an intercept and a random slope. The time between inclusion and the date of the visit will be included in the model in weeks. This analysis will be performed independently for the following parameters as dependent variables:

- Patients' weight before dialysis at each follow-up visit
- Patients' weight after dialysis at each follow-up visit
- Weight lost during dialysis
- Δ weight visit

10.2.3.2. Change in blood pressure

Blood pressure changes: The average BP measurements and the delta between measurements before and after dialysis will be presented at inclusion and at each period by exposure group independently in the patient population and the exposed-unexposed population.
The change in blood pressure over time will also be modelled using a mixed linear model as the primary endpoint detailed in section 8.1 and compared between the two exposure groups after adjustment for the parameters listed in section 7.7 .

10.2.4 Hospitalisations and other complications

1. The number and percentage of subjects hospitalised at least once during the study period will be presented. The number of hospitalisations, duration (%) in each department and total duration (%) during the study will be described in each exposure group, independently in the patient population and the exposed-unexposed population.

   The rate and time to onset of the first hospitalisation will be estimated using the Kaplan-Meier method independently in each of the exposure groups.

   The percentage of *total hospitalisation duration* will be compared between the two exposure groups using a generalised linear mixed model with a beta distribution and a logit function. The percentage will need to be divided by 100 for the model, and to avoid problems with the values 0.00 and 1.00, these will be replaced by 0.0001 and 0.9999 respectively.

   In addition, the ratio of the number of days of hospitalisation will be described in each exposure group.

2. Cardiovascular complications (see §7.4.4):

   The frequency and percentage of patients with occurrence of at least one cardiovascular complication will be presented by exposure group. Complications will also be grouped by *System Organ Classes* (SOC) and *Preferred Terms* (PT) and will be presented by exposure group independently in the patient population and the exposed-unexposed population.

   If at least 5 patients have experienced one or more cardiovascular events, the occurrence of a cardiovascular complication in the study will be estimated using the Kaplan-Meier method to estimate the survival function in the patient population over the entire study period and

independently in each of the exposure groups. The two exposure groups will be compared using a Cox model adjusted for the parameters listed in §7.7 .

3. The frequency and percentage of subjects with at least one surgical procedure will be presented in each exposure group, independently in the patient population and the exposed-unexposed population.
4. The frequency and percentage of subjects who died during the study will be presented in each exposure group in the patient population and the exposed-unexposed population.

10.2.5 Self-administered questionnaires

1. The Kidney Disease Quality of Life Instrument (KDQOL-SF™) questionnaire scores will be described by exposure group at each visit. The change in the overall score will be analysed using a generalised linear mixed model adjusted for the parameters listed in §7.7.
2. Changes in sleep duration over time will be analysed using a linear regression model with repeated data, adjusted for the exposure group as a fixed effect and the measurement time since inclusion as a random effect, in the wristband subgroup.
3. The percentage of patients with RLS will be presented by exposure group independently in the exposed and unexposed patient populations. The percentages of subjects with RLS at inclusion and at the end of the study will be compared independently using a chi-square test.

   The time to onset of RLS will be estimated in the subgroup of patients with no RLS documented at inclusion (if at least 50% of subjects do not have RLS at inclusion) using an actuarial method, per quarter in each group.

   Data from the IRLS (International Restless Legs Syndrome Rating Scale) questionnaire and derived variables will be described in the RLS subgroup by exposure group, independently in the patient population and the exposed-unexposed population.

11. ANALYSIS OF VIGILANCE PARAMETERS

11.1. Adverse Event

Adverse events (including cardiovascular complications) will be presented by *System Organ Class* (SOC) and *Preferred Term* (PT), in alphabetical order, by exposure group in the vigilance population.
The following will also be presented by exposure group in the vigilance population:

- Frequency and percentage of patients experiencing at least one adverse event
- Frequency and percentage of patients experiencing at least one serious adverse event

All AEs/SAEs will be presented in the form of listings using the 5 levels of the MedDRA dictionary. Only patients with at least one AE will be included in the listings.

11.2. Vital Signs and Clinical Examinations

Blood pressure analysis is detailed in §10.2.3.2 .

11.3. Laboratory Parameters

Biochemical data will be described at inclusion and by visit period by exposure group in the patient population and the exposed-unexposed population.

11.4. Other Analyses

11.4.1 Physical activity and lockdown

To estimate the possible influence of confinement on physical activity, physical activity during confinement will be analysed in the Lockdown subgroup. This analysis will only be performed if data are available for at least 20 patients. Data on chronological age, gender, length of dialysis and physical activity parameters (see §7.6.1) will be described by exposure group. In addition, the Prochaska and DiClemente stages will be cross-referenced with the lockdown period * outcome of lockdown by exposure group.

The deltas will be compared between the two groups using a generalised mixed model adjusted for the covariates listed in §7.7. The delta will be the dependent variable and the exposure group and period (during lockdown/post-lockdown) will be entered as fixed effects in the model. If a delta does not follow a normal distribution, the estimated tertiles for the lockdown subgroup will be used for the analysis.

11.4.2 Characteristics of follow-up visits

The frequency and percentage of patients who attended the visit (1 month, 2 months, etc.) will be presented for each visit in the patient population and the exposed/unexposed population.

11.4.3 Premature withdrawal from the study

Data from the end of study form will be described in each exposure group and independently in the patient population and the exposed-unexposed population.

In addition, the percentage of subjects who permanently discontinued the study during the follow-up period will be estimated using the Kaplan-Meier method for the entire population and independently in each of the exposure groups. This estimate will be made for the patient population and the ENE population.

Subjects who did not withdraw will be censored on the date of last contact, date of death or date of points, whichever occurred first.

12. RISK ASSESSMENT

A review of the data will be prepared by the data manager, which will enable the quality of the data to be used for statistical analysis to be estimated.

To validate the statistical analysis, a second statistician will check the results and the corresponding SAS log to detect any unexpected errors or warnings in the log or any discrepancies in the results. If necessary, corrections will be made and verification will be performed as many times as necessary.

13. ANALYSIS TABLES AND FIGURES

TABLE LISTS

Table 1. Patient Dispositions

Table 2. Inclusion/exclusion criteria and major deviations from the protocol

Table 3. Dates of initial informed consent, initial inclusion, and final visit

Table 4. Centre characteristics

Inclusion data

Unless otherwise specified, data will be presented by exposure group

Table 5. Socio-demographic data (patient population)

Table 6. Blood pressure (patient population)

Table 7. Characteristics of chronic renal failure (patient population)

Table 8. Comorbidities (patient population)

Table 9. Medication treatment on the day of inclusion (patient population)

Table 10. Self-administered questionnaires / Connected wristband / Patient diary (patient population)

Table 11. Blood tests (quantitative data, patient population)

Table 12. Blood tests (interpretation of results, patient population)

Table 13. Urinary tesst (patient population)

Table 14. Dialysis characteristics (patient population)

Table 15. Prescription at the end of the consultation (patient population)

Table 16. Socio-demographic data (ENE population)

Table 17. Blood pressure (ENE population)

Table 18. Chronic failure characteristics (ENE population)

Table 19. Comorbidities (ENE population)

Table 20. Medication treatment on the day of inclusion (ENE population)

Table 21. Self-administered questionnaires / Connected wristband / Patient diary (ENE population)

Table 22. Characteristics of dialysis in a centre (ENE population)

Table 23. Blood tests (quantitative data, ENE population)

Table 24. Blood tests (interpretation of results, ENE population)

Table 25. Urinary tests (ENE population)

Table 26. Characteristics of dialysis (ENE population)

Table 27. Prescription at the end of the consultation (ENE population)

Monthly data

Unless otherwise specified, data will be presented by exposure group per monthly visit V1, V2, V4, V5, V7, V8, V10 and V11

Table 28. Characteristics of monthly visits (patient population)

Table 29. Characteristics of monthly visits (ENE population)

Table 30. Clinical data (Patient population)

Table 31. Clinical data (ENE population)
Table 32. Blood pressure (Patient population)
Table 33. Blood pressure (ENE population)
Table 34. Dialysis characteristics (Patient population)
Table 35. Dialysis characteristics (ENE population)
Table 36. Drug treatment (Patient population)
Table 37. Drug treatment (ENE population)
Table 38. Blood tests (quantitative data, patient population)
Table 39. Blood tests (interpretation of results, patient population)
Table 40. Urinary tests (patient population)
Table 41. Blood tests (quantitative data, ENE population)
Table 42. Blood tests (interpretation of results, ENE population)
Table 43. Urinary analysis (ENE population)

Quarterly data

Unless otherwise specified, data will be presented by exposure group and by quarterly visit V3, V6, V9 and V12

Table 44. Quarterly visit information
Table 45. Clinical data
Table 46. Blood pressure
Table 47. Dialysis characteristics
Table 48. In-centre dialysis characteristics (non-exposed subgroup)
Table 49. Home dialysis characteristics (exposed subgroup)
Table 50. Drug treatment
Table 51. Blood tests (quantitative data, patient population)
Table 52. Blood tests (interpretation of results, patient population)
Table 53. Urinary tests (patient population)
Table 54. Blood tests (quantitative data, ENE population)
Table 55. Blood test (interpretation of results, ENE population)
Table 56. Urinary tests (ENE population)

Half-yearly data

Unless otherwise specified, data will be presented by exposure group in the ENE population at the inclusion visit, V6 and V12

Table 57. KDQL questionnaire data (ENE population)
Table 58. Patient diary data (ENE population)
Table 59. End of study data (ENE population)
Table 60. Daily haemodialysis discontinuation data (exposed subgroup, ENE population)
Table 61. Data on cessation of haemodialysis in centres (non-exposed subgroup, ENE population)

Analysis of the primary endpoint

Table 62. Number of steps/day (ENE population)

Table 63. Analysis of the average total number of steps (univariate analysis, ENE population)

Table 64. Analysis of the average total number of steps (multivariate analysis, ENE population)

Table 65. Analysis of the average total number of steps (Interaction test, ENE population)

Table 66. Analysis of the average total number of steps (final model, ENE population)

Other physical activity and sleep data

Table 67. Secondary physical activity criteria (wristband data, patient population)

Table 68. Change in average daily sedentary time (patient population)

Table 69. Change in number of steps on days with and without dialysis (HCC group, patient population)

Table 70. Prochaska and diClemente stage and physical activity (supplementary questionnaire, patient population)

Table 71. Prochaska and diClemente stage at inclusion and at the end of the study (supplementary questionnaire, HHD group, patient population)

Table 72. Prochaska and diClemente stage at inclusion and at the end of the study (supplementary questionnaire, HCC group, patient population)

Table 73. Sleep (wristband data, patient population)

Table 74. Change in sleep duration over time (patient population)

Table 75. Sleep (wristband data, ENE population)

Table 76. Change in sleep duration over time (ENE population)

Changes in weight and blood pressure

Linear regression models for repeated data

Table 77. Changes in patient weight before dialysis at each follow-up visit (ENE population)

Table 78. Change in dialysis weight delta (ENE population)

Table 79. Changes in weight delta between the first and last visits in the study (ENE population)

Table 80. Changes in blood pressure during the study (ENE population)

Complications and adverse effects

Table 81. Dropout rate (Kaplan Meier method, HHD subjects)

Table 82. Hospitalisation and other complications (patient population)

Table 83. Cardiovascular events (patient population)

Table 84. Rate and time to onset of cardiovascular complications (patient population)

Table 85. Hospitalisation and other complications (ENE population)

Table 86. Cardiovascular events (ENE population)

Table 87. Rate and time to onset of cardiovascular complications (ENE population)

Table 88. Adverse events (AEs) (vigilance population)

Table 89. Serious adverse events (SAEs) (vigilance population)

Table 90. Adverse events related to the connected wristband (vigilance population)

Lockdown

Table 91. Physical activity during lockdown (lockdown population)
Table 92. Physical activity related to lockdown (lockdown population)
Table 93. Prochaska and DiClemente stages during and after lockdown (HHD subgroup)
Table 94. Prochaska and DiClemente stages during and after lockdown (HCC subgroup)
Table 95. Changes in physical activity during lockdown (lockdown population)

LIST OF FIGURES

Figure 1. Geographical distribution of centres in France
Figure 2. Patient distribution
Figure 3. Distribution of subjects by sex (patient population)
Figure 4. Distribution of subjects by exposure groups
Figure 5. Survival curves for the occurrence of first cardiovascular complication (patient population)
Figure 6. Survival curves for the occurrence of first cardiovascular complication (ENE population)
Figure 7. Survival curves for dropout rates (Kaplan Meier, HHD patients)
Figure 8. Kidney transplants (Kaplan Meier, patient population)
Figure 9. Weight change before dialysis by exposure group (patient population)
Figure 10. Blood pressure changes before dialysis by exposure group (patient population)
Figure 11. Changes in blood calcium levels by exposure group
Figure 12. Change in average number of daily steps by exposure group (patient population)
Figure 13. Change in average number of daily steps by exposure group (ENE population)
Figure 14. Change in average daily sedentary time by exposure group (patient population)
Figure 15. Change in average daily sedentary time by exposure group (ENE population)
Figure 16. Change in average sleep duration by exposure group (patient population)
Figure 17. Change in average sleep duration by exposure group (ENE population)
Figure 18. Severity of SJRS at inclusion, 6 months and end of study by exposure group (patient population)
Figure 19. Severe sleep apnoea syndrome severity at inclusion, 6 months and end of study by exposure group (ENE population).

Additional figures may be edited if necessary to clarify the results

LISTING

Listing 1. Demographic characteristics
Listing 2. Hospitalisations
Listing 3. Surgical procedures

Listing 4. Drug treatments
Listing 5. Adverse events
Listing 6. Serious adverse events
Listing 7. Withdrawal from study
Listing 8. Discontinuation of daily haemodialysis

14. REFERENCES

[1] Avesani CM, Trolonge S, Deléaval P, Baria F, Mafra D, Faxén-Irving G, Chauveau P, Teta D, Kamimura MA, Cuppari L, Chan M, Heimbürger O, Fouque D; Physical activity and energy expenditure in haemodialysis patients: an international survey. Nephrol Dial Transplant. 2012;27(6):2430-4.

[2] Carvalho EV, Reboredo MM, Gomes EP, Teixeira DR, Roberti NC, Mendes JO, Oliveira JCA, Sanders-Pinheiro H, Pinheiro BV; Physical Activity in Daily Life Assessed by an Accelerometer in Kidney Transplant Recipients and Haemodialysis Patients. Transplantation Proceedings. 2014; 46(6):1713-7

[3] World Health Organisation. BMI classification. Available at: https://www.euro.who.int/en/health-topics/disease-prevention/nutrition/a-healthy-lifestyle/body-mass-index-bmi. Last accessed 26/08/2021

[4] WHO Drug ATC code dictionary. Available at: https://www.whocc.no/atc_ddd_index/ Last accessed 30/08/2021

[5] Marcus BH, Simkin LR. The stages of exercise behaviour. J Sports Med Phys Fitness. 1993 Mar;33(1):83-8.

[6] ANSM: Directory of pharmaceutical specialities enoxaparin http://agence-prd.ansm.sante.fr/php/ecodex/index.php#result. Last accessed 27/09/2021

[7] Hays, R.D., et. al. (1997). Kidney Disease Quality of Life Short Form (KDQOL-SF™), Version 1.3: A Manual for Use and Scoring. Santa Monica, CA: RAND, P-7994. Available at: https://www.rand.org/health-care/surveys_tools/kdqol.html. Last accessed 26/08/2021

[8] Walters AS, LeBrocq C, Dhar A, Hening W, Rosen R, Allen RP, Trenkwalder C; International Restless Legs Syndrome Study Group. Validation of the International Restless Legs Syndrome Study Group rating scale for restless legs syndrome. Sleep Med. 2003;4(2):121-32

Supplemental Figure 1 - Discontinuation of Short-Daily Home Hemodialysis (FAS)

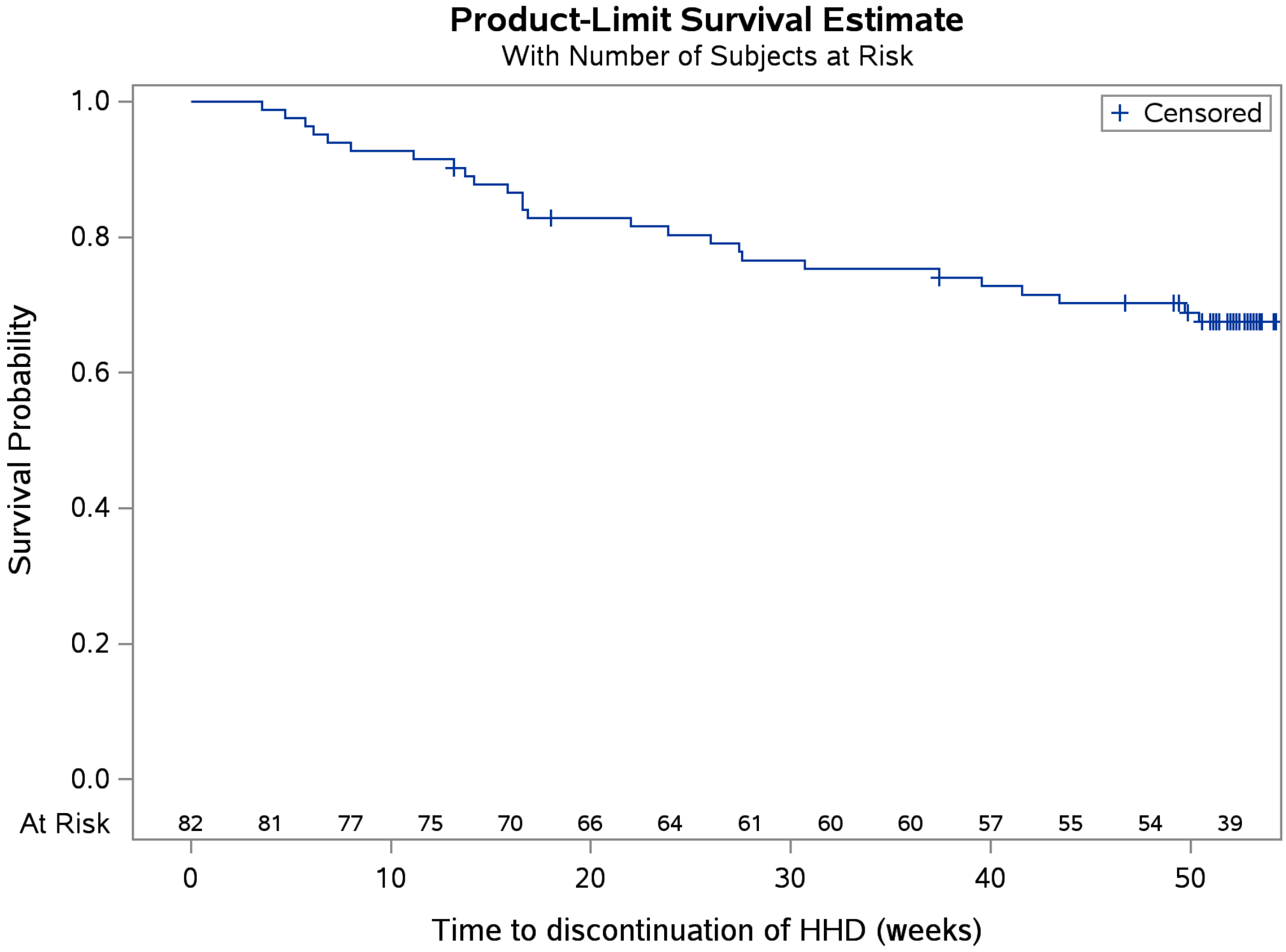

FAS, Full Analysis Set; HHD, short-daily Home HemoDialysis

Supplemental Figure 2 - Kidney Transplant (FAS)

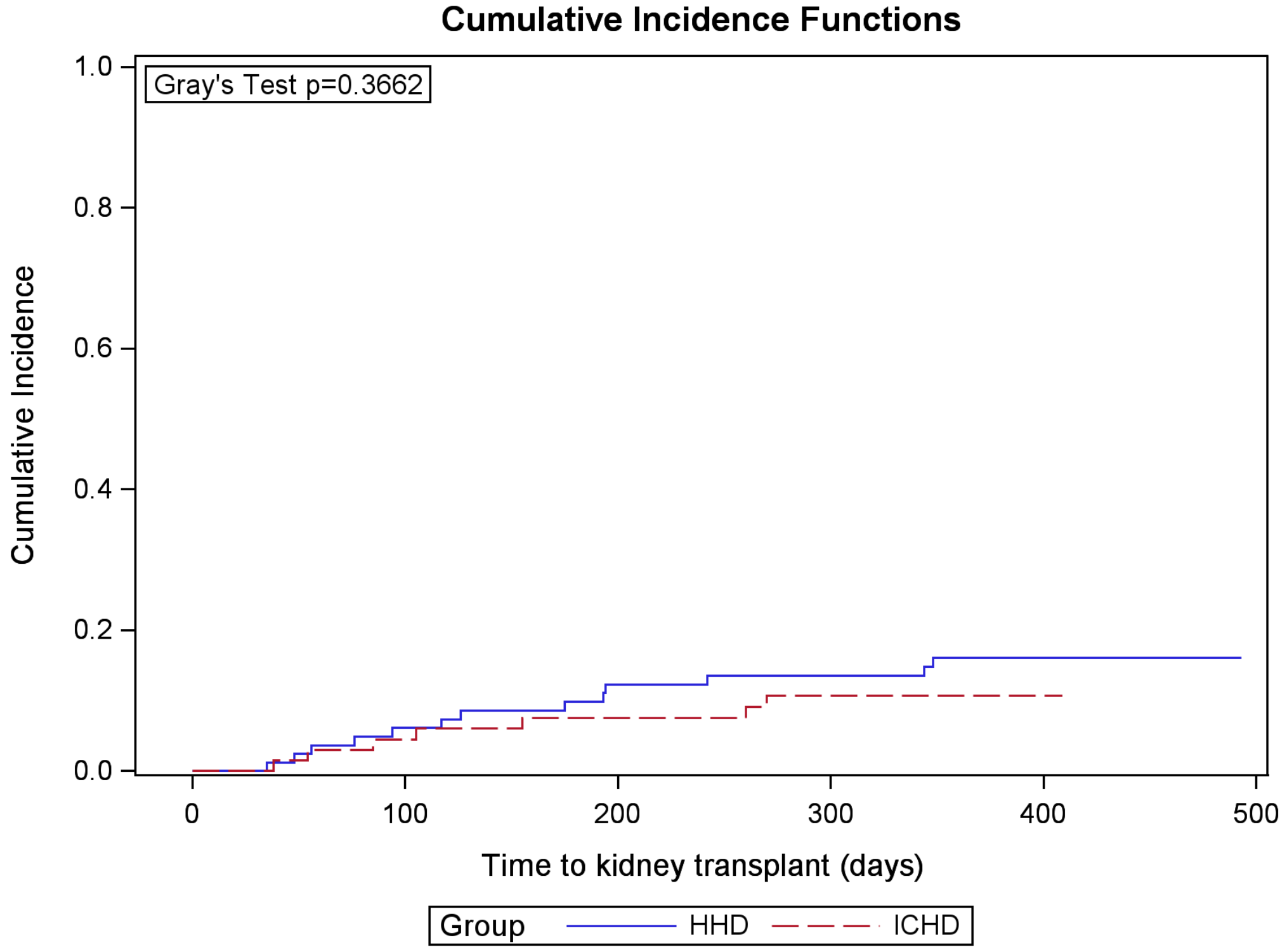

FAS, Full Analysis Set; HHD, short-daily Home HemoDialysis; ICHD, In-Center HemoDialysis

Supplemental Figure 3 - Mean Average Daily Steps in Patients with no Chronic Heart Failure

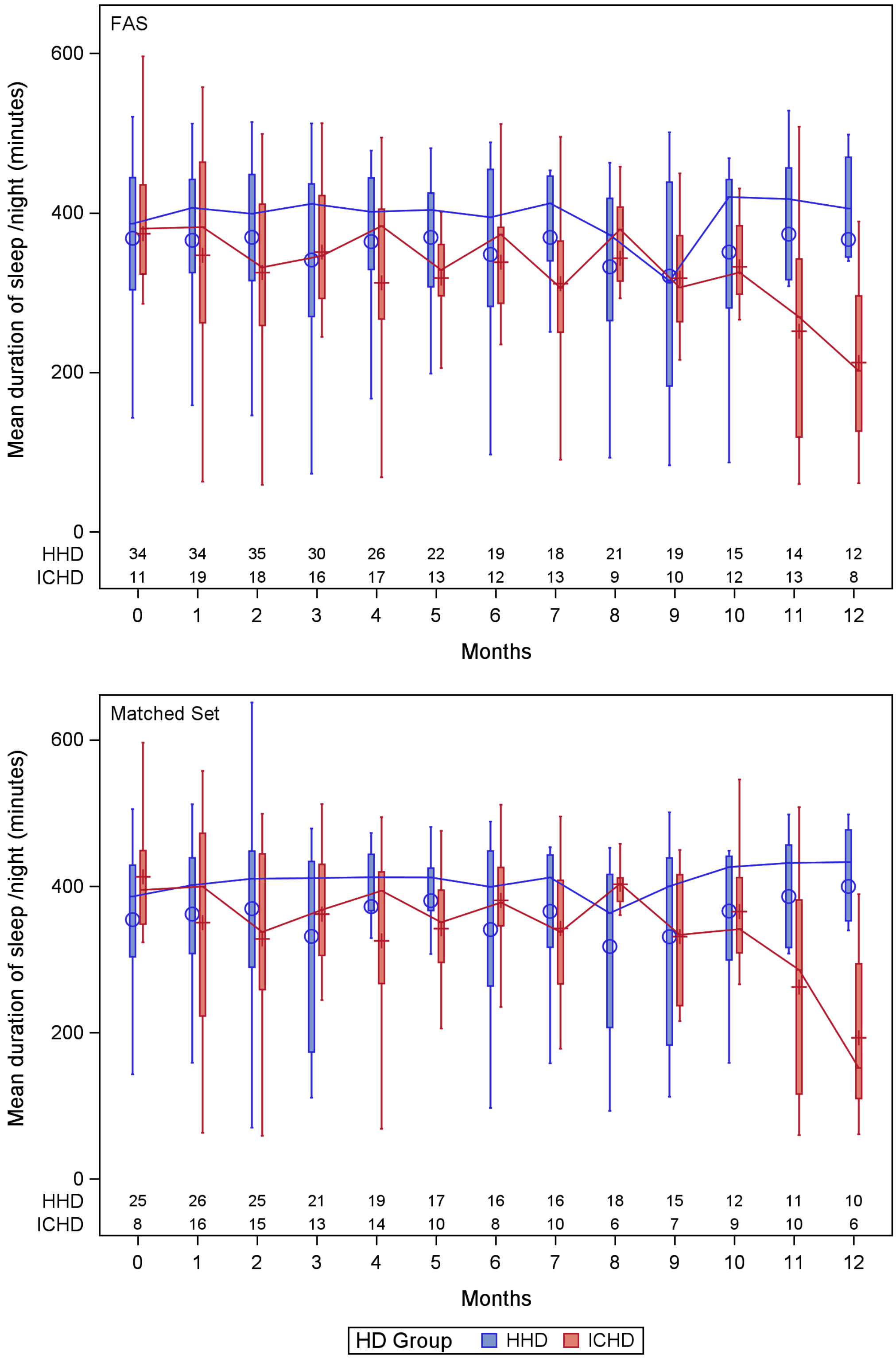

FAS, Full Analysis Set; HHD, short-daily Home HemoDialysis; ICHD, In-Center HemoDialysis

Supplemental Figure 4 - Body Weight After Dialysis

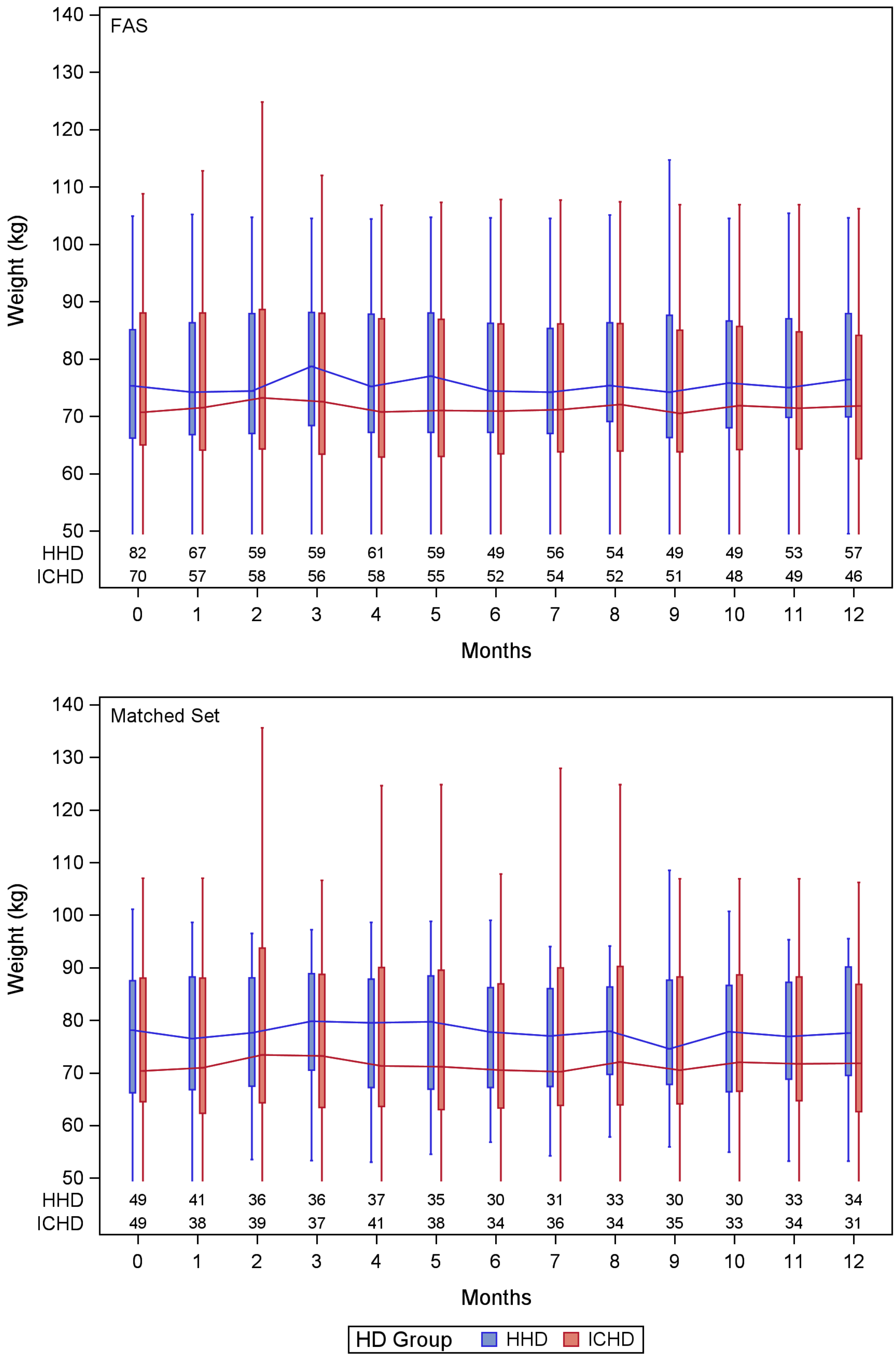

FAS, Full Analysis Set; HHD, short-daily Home HemoDialysis; ICHD, In-Center HemoDialysis

Supplemental Table 1. KDQOL scores (FAS)

	Inclusion		6 Months		12 Months	
	HHD	ICHD	HHD	ICHD	HHD	ICHD
**Symptoms–Problems**						
n	73	54	33	33	36	30
Médiane [first quartile; third quartile]	75.0 [62.5 ; 85.4]	81.3 [72.9 ; 87.5]	81.3 [70.8 ; 84.1]	75.0 [66.7 ; 81.3]	82.6 [75.0 ; 88.5]	76.0 [62.5 ; 83.3]
**Effects of Kidney Disease**						
n	73	54	33	34	35	29
Médiane [first quartile; third quartile]	65.6 [43.8 ; 78.1]	63.4 [43.8 ; 81.3]	65.6 [50.0 ; 84.4]	56.7 [37.5 ; 68.8]	75.0 [53.1 ; 87.5]	62.5 [46.9 ; 75.0]
**Burden of Kidney Disease**						
n	71	55	33	34	36	30
Médiane [first quartile; third quartile]	43.8 [25.0 ; 56.3]	43.8 [25.0 ; 56.3]	56.3 [37.5 ; 75.0]	37.5 [25.0 ; 50.0]	56.3 [28.1 ; 75.0]	37.5 [18.8 ; 50.0]
**Work Status**						
n	69	52	32	30	34	30
Médiane [first quartile; third quartile]	50.0 [0.0 ; 100.0]	0.0 [0.0 ; 50.0]	50.0 [0.0 ; 100.0]	0.0 [0.0 ; 50.0]	50.0 [0.0 ; 100.0]	0.0 [0.0 ; 50.0]
**Cognitive Function**						
n	71	53	33	34	36	30
Médiane [first quartile; third quartile]	80.0 [66.7 ; 93.3]	80.0 [66.7 ; 86.7]	86.7 [66.7 ; 93.3]	76.7 [60.0 ; 86.7]	90.0 [66.7 ; 100.0]	73.3 [66.7 ; 93.3]
**Quality of Social Interaction**						
n	71	53	33	34	36	30
Médiane [first quartile; third quartile]	80.0 [66.7 ; 93.3]	80.0 [66.7 ; 86.7]	80.0 [60.0 ; 93.3]	80.0 [73.3 ; 93.3]	80.0 [66.7 ; 93.3]	83.3 [73.3 ; 100.0]
**Sexual Function**						
n	70	44	33	30	33	25
Médiane [first quartile; third quartile]	75.0 [50.0 ; 100.0]	56.3 [25.0 ; 87.5]	75.0 [37.5 ; 100.0]	75.0 [50.0 ; 100.0]	75.0 [25.0 ; 100.0]	75.0 [25.0 ; 100.0]
**Sleep**						
n	68	53	32	33	36	30
Médiane [first quartile; third quartile]	70.0 [50.0 ; 82.5]	60.0 [42.5 ; 67.5]	62.5 [50.0 ; 78.8]	50.0 [35.0 ; 62.5]	68.8 [55.8 ; 81.3]	52.5 [40.0 ; 72.5]
**Social Support**						
n	67	52	32	33	36	29
Médiane [first quartile; third quartile]	83.3 [66.7 ; 83.3]	66.7 [58.3 ; 83.3]	75.0 [58.3 ; 83.3]	66.7 [33.3 ; 83.3]	66.7 [58.3 ; 83.3]	66.7 [50.0 ; 83.3]
**Dialysis Staff Encouragement**						
n	64	51	32	29	36	25

	Inclusion		6 Months		12 Months	
	HHD	ICHD	HHD	ICHD	HHD	ICHD
Médiane [first quartile; third quartile]	100.0 [87.5 ; 100.0]	100.0 [75.0 ; 100.0]	100.0 [93.8 ; 100.0]	87.5 [75.0 ; 100.0]	100.0 [75.0 ; 100.0]	87.5 [75.0 ; 100.0]
**Patient Satisfaction Rating**						
n	64	51	32	29	36	25
Médiane [first quartile; third quartile]	83.3 [83.3 ; 100.0]	83.3 [83.3 ; 100.0]	100.0 [83.3 ; 100.0]	83.3 [66.7 ; 100.0]	83.3 [83.3 ; 100.0]	83.3 [66.7 ; 100.0]
**Physical Functioning**						
n	73	53	33	34	36	30
Médiane [first quartile; third quartile]	80.0 [60.0 ; 90.0]	85.0 [65.0 ; 90.0]	75.0 [65.0 ; 90.0]	70.0 [45.0 ; 90.0]	77.5 [62.5 ; 92.5]	67.5 [45.0 ; 85.0]
**Role Limitations--Physical**						
n	73	55	33	34	36	30
Médiane [first quartile; third quartile]	25.0 [0.0 ; 75.0]	25.0 [0.0 ; 75.0]	100.0 [25.0 ; 100.0]	25.0 [0.0 ; 50.0]	75.0 [37.5 ; 100.0]	25.0 [0.0 ; 100.0]
**Pain**						
n	73	55	33	34	36	30
Médiane [first quartile; third quartile]	67.5 [45.0 ; 90.0]	60.0 [45.0 ; 90.0]	67.5 [45.0 ; 100.0]	56.3 [45.0 ; 90.0]	83.8 [45.0 ; 100.0]	62.5 [45.0 ; 77.5]
**General Health**						
n	73	54	33	34	36	30
Médiane [first quartile; third quartile]	50.0 [30.0 ; 60.0]	47.5 [30.0 ; 60.0]	55.0 [30.0 ; 65.0]	42.5 [35.0 ; 55.0]	55.0 [40.0 ; 67.5]	50.0 [35.0 ; 65.0]
**Emotional Well-Being**						
n	73	55	33	34	35	30
Médiane [first quartile; third quartile]	68.0 [52.0 ; 80.0]	68.0 [52.0 ; 80.0]	72.0 [52.0 ; 88.0]	60.0 [48.0 ; 80.0]	72.0 [56.0 ; 84.0]	64.0 [52.0 ; 80.0]
**Role Limitations--Emotional**						
n	73	55	33	33	36	30
Médiane [first quartile; third quartile]	66.7 [0.0 ; 100.0]	66.7 [0.0 ; 100.0]	100.0 [33.3 ; 100.0]	33.3 [0.0 ; 100.0]	100.0 [50.0 ; 100.0]	33.3 [0.0 ; 100.0]
**Social Function**						
n	73	55	33	34	36	30
Médiane [first quartile; third quartile]	62.5 [50.0 ; 87.5]	75.0 [50.0 ; 100.0]	87.5 [62.5 ; 100.0]	62.5 [37.5 ; 87.5]	87.5 [50.0 ; 100.0]	62.5 [50.0 ; 100.0]
**Energy/Fatigue**						
n	73	55	33	34	35	30
Médiane [first quartile; third quartile]	45.0 [30.0 ; 60.0]	45.0 [40.0 ; 65.0]	60.0 [45.0 ; 70.0]	45.0 [35.0 ; 60.0]	55.0 [45.0 ; 70.0]	50.0 [35.0 ; 55.0]
**Overall Health Rating**						
n	69	53	32	30	34	30
Médiane [first quartile; third quartile]	60.0 [50.0 ; 70.0]	60.0 [40.0 ; 70.0]	65.0 [50.0 ; 80.0]	55.0 [50.0 ; 70.0]	70.0 [50.0 ; 70.0]	50.0 [40.0 ; 70.0]

n documented patients. FAS, full analysis set; HD, hemodialysis; HHD, home short-daily hemodialysis; ICHD, in-center hemodialysis. KDQOL French version 1.2

Supplemental Table 2. Predialysis Weight and Blood Pressure during the study (FAS)

Item / Month	HD Modality	0	1	2	3	4	5
Body Weight	**HHD**	75.7 [67.7 ; 85.9]	75.0 [68.0 ; 87.3]	75.9 [67.7 ; 88.8]	77.6 [69.8 ; 88.5]	76.2 [67.8 ; 88.2]	77.5 [68.2 ; 89.7]
	**ICHD**	73.0 [67.6 ; 90.0]	73.7 [64.7 ; 91.1]	74.8 [65.7 ; 91.1]	73.8 [66.5 ; 89.3]	73.6 [64.7 ; 89.2]	73.3 [65.7 ; 88.8]
Systolic Blood Pressure	**HHD**	138.8 [127.5 ; 153.8]	143.3 [133.0 ; 153.0]	138.8 [127.0 ; 151.3]	143.8 [130.3 ; 156.8]	139.7 [124.0 ; 151.3]	142.0 [129.0 ; 152.7]
	**ICHD**	139.7 [127.7 ; 150.0]	140.0 [125.3 ; 152.3]	136.5 [128.8 ; 149.3]	142.3 [128.3 ; 152.0]	139.0 [125.3 ; 156.0]	141.7 [133.0 ; 159.5]
Diastolic Blood Pressure	**HHD**	80.3 [72.5 ; 87.6]	79.3 [73.3 ; 90.7]	80.3 [71.7 ; 86.3]	79.6 [73.2 ; 86.5]	77.0 [73.0 ; 84.7]	80.3 [73.0 ; 88.0]
	**ICHD**	78.3 [67.7 ; 85.0]	79.3 [69.0 ; 85.3]	76.7 [66.8 ; 85.3]	77.3 [72.0 ; 89.7]	77.8 [68.0 ; 89.3]	82.8 [69.5 ; 89.5]

Item / Month	HD Modality	6	7	8	9	10	11	12
Body Weight	**HHD**	75.8 [67.6 ; 88.2]	74.0 [67.1 ; 86.7]	78.0 [69.2 ; 88.8]	75.3 [66.9 ; 88.7]	76.5 [67.7 ; 87.8]	76.6 [69.4 ; 89.1]	76.7 [69.0 ; 89.1]
	**ICHD**	72.5 [64.9 ; 87.9]	72.6 [65.4 ; 87.6]	73.5 [65.9 ; 88.1]	72.1 [66.0 ; 87.6]	74.3 [66.5 ; 88.2]	72.9 [65.5 ; 86.6]	73.7 [65.1 ; 86.1]
Systolic Blood Pressure	**HHD**	143.3 [131.0 ; 153.0]	142.0 [129.0 ; 153.0]	135.7 [122.5 ; 151.7]	139.0 [126.8 ; 151.5]	139.7 [130.0 ; 152.3]	147.7 [130.7 ; 160.5]	144.0 [130.0 ; 156.0]
	**ICHD**	141.7 [125.0 ; 157.0]	141.0 [128.3 ; 154.5]	138.7 [121.7 ; 153.7]	141.0 [124.7 ; 156.7]	137.5 [128.2 ; 154.8]	135.2 [126.7 ; 150.7]	143.0 [124.7 ; 154.7]
Diastolic Blood Pressure	**HHD**	80.7 [70.7 ; 87.7]	80.0 [71.0 ; 90.0]	77.0 [68.0 ; 88.7]	82.7 [68.6 ; 90.0]	78.3 [69.0 ; 86.3]	81.5 [72.2 ; 88.5]	81.3 [76.0 ; 87.7]
	**ICHD**	78.7 [70.0 ; 85.0]	80.8 [67.7 ; 89.0]	80.0 [65.7 ; 87.0]	80.7 [70.3 ; 87.3]	80.0 [71.0 ; 88.8]	76.5 [70.0 ; 86.0]	78.0 [70.0 ; 88.0]

Median [first quartile; third quartile]

Supplemental Table 3. Blood Laboratory Data (FAS)

Item / Month	HD Modality	0	1	2	3	4	5	6
Number of subjects	**HHD**	81	70	64	61	63	60	55
	**ICHD**	69	57	59	56	57	52	52
S-Phosphate (mmol/L)	**HHD**	1.5 [1.3 ; 1.9]	1.4 [1.2 ; 1.7]	1.5 [1.3 ; 1.9]	1.5 [1.2 ; 1.8]	1.5 [1.4 ; 1.9]	1.6 [1.3 ; 1.8]	1.6 [1.3 ; 1.9]
	**ICHD**	1.6 [1.3 ; 2.0]	1.6 [1.2 ; 2.0]	1.6 [1.3 ; 1.9]	1.6 [1.2 ; 2.0]	1.6 [1.1 ; 1.9]	1.5 [1.2 ; 1.8]	1.6 [1.3 ; 2.0]
S-PTH (pg/mL)	**HHD**	343 [196 ; 538]	306 [213 ; 633]	324 [212 ; 627]	347 [199 ; 633]	373 [193 ; 601]	415 [203 ; 566]	354 [195 ; 555]
	**ICHD**	358 [193 ; 592]	299 [203 ; 556]	293 [198 ; 498]	261 [191 ; 511]	404 [233 ; 781]	406 [209 ; 711]	366 [209 ; 536]
S-Calcium (mmol/L)	**HHD**	2.3 [2.2 ; 2.4]	2.2 [2.1 ; 2.3]	2.3 [2.1 ; 2.4]	2.2 [2.1 ; 2.4]	2.2 [2.1 ; 2.4]	2.3 [2.1 ; 2.4]	2.2 [2.1 ; 2.4]
	**ICHD**	2.2 [2.1 ; 2.3]	2.2 [2.1 ; 2.3]	2.2 [2.1 ; 2.3]	2.2 [2.1 ; 2.3]	2.2 [2.1 ; 2.3]	2.2 [2.1 ; 2.3]	2.2 [2.1 ; 2.3]
S-Alkakline Phosphatase (IU/L)	**HHD**	80.0 [62.0 ; 110.0]	88.0 [65.0 ; 119.0]	79.0 [62.0 ; 117.0]	85.0 [63.0 ; 103.5]	76.0 [67.0 ; 104.0]	82.0 [69.0 ; 108.0]	82.0 [66.0 ; 139.0]
	**ICHD**	85.0 [72.0 ; 120.0]	82.0 [65.0 ; 128.0]	83.5 [71.0 ; 121.0]	80.0 [59.0 ; 118.0]	77.5 [70.0 ; 116.0]	102.5 [78.0 ; 138.0]	98.5 [68.0 ; 125.0]
Na (mmol/L)	**HHD**	139 [137 ; 140]	139 [138 ; 140]	139 [138 ; 141]	139 [137 ; 140]	138 [137 ; 140]	139 [137 ; 140]	138 [137 ; 140]
	**ICHD**	138 [136 ; 140]	137 [136 ; 140]	139 [137 ; 140]	138 [137 ; 140]	138 [136 ; 140]	139 [136 ; 141]	138 [136 ; 140]
HCO3 (mmol/L)	**HHD**	22 [20 ; 24]	23 [21 ; 25]	23 [21 ; 24]	22 [20 ; 24]	23 [21 ; 25]	23 [21 ; 24]	22 [21 ; 24]
	**ICHD**	22 [20 ; 24]	23 [21 ; 25]	23 [20 ; 24]	23 [22 ; 25]	23 [21 ; 24]	23 [21 ; 25]	23 [22 ; 25]
Number of subjects (Hemoglobin)	**HHD**	79	69	66	62	64	61	57
	**ICHD**	69	56	59	56	57	54	52
Hemoglobin (g/dL)	**HHD**	11.1 [10.4 ; 12.3]	10.3 [9.4 ; 11.2]	10.2 [9.2 ; 11.4]	10.7 [9.4 ; 11.6]	10.6 [10.0 ; 11.6]	10.5 [10.0 ; 11.6]	10.4 [9.3 ; 11.3]
	**ICHD**	11.2 [10.4 ; 12.4]	11.4 [10.5 ; 12.4]	11.3 [10.3 ; 12.6]	11.3 [10.2 ; 12.3]	11.5 [10.3 ; 12.3]	11.2 [10.3 ; 11.8]	11.3 [10.4 ; 11.8]
Number of subjects (S-Ferritin)	**HHD**	75	57	57	52	51	45	45
	**ICHD**	60	42	48	44	47	43	42
S-Ferritin (µg/L)	**HHD**	201 [117 ; 320]	199 [104 ; 359]	149 [78 ; 395]	142 [48 ; 286]	143 [42 ; 306]	112 [68 ; 331]	101 [40 ; 202]
	**ICHD**	356 [216 ; 496]	314 [206 ; 506]	332 [197 ; 393]	325 [201 ; 570]	407 [209 ; 580]	384 [226 ; 576]	336 [222 ; 568]
Transferrin Saturation (%)	**HHD**	21 [17 ; 28]	21 [17 ; 29]	18 [13 ; 25]	19 [14 ; 25]	19 [14 ; 26]	20 [13 ; 28]	15 [10 ; 24]
	**ICHD**	29 [21 ; 39]	25 [18 ; 33]	23 [18 ; 33]	24 [19 ; 29]	26 [21 ; 32]	25 [19 ; 33]	25 [19 ; 31]
Albumin (g/L)	**HHD**	39 [37 ; 43]	41 [37 ; 44]	42 [38 ; 45]	39 [37 ; 44]	40 [38 ; 44]	40 [38 ; 44]	40 [37 ; 43]
	**ICHD**	39 [36 ; 41]	38 [36 ; 42]	39 [35 ; 41]	39 [37 ; 43]	40 [35 ; 42]	38 [35 ; 42]	39 [37 ; 42]

Item / Month	HD Modality	0	1	2	3	4	5	6
Predialysis S-B2M (mg/L)	**HHD**	17.6 [14.7 ; 28.3]	18.3 [15.4 ; 22.5]	18.4 [17.9 ; 20.9]	16.3 [13.5 ; 18.5]	18.7 [13.2 ; 25.5]	15.9 [11.5 ; 19.3]	16.8 [15.2 ; 20.9]
	**ICHD**	22.5 [17.5 ; 27.2]	24.4 [18.7 ; 28.2]	24.6 [16.8 ; 31.5]	20.5 [17.6 ; 26.8]	22.5 [16.9 ; 27.5]	19.7 [15.6 ; 31.4]	26.6 [15.3 ; 30.0]
Hb1Ac (%)	**HHD**	5.7 [5.1 ; 6.1]	5.6 [5.2 ; 6.9]	5.3 [4.6 ; 6.4]	5.0 [4.3 ; 5.5]	5.1 [4.8 ; 5.6]	5.8 [5.2 ; 7.7]	5.4 [5.0 ; 5.9]
	**ICHD**	5.4 [5.1 ; 5.9]	5.1 [4.7 ; 9.1]	5.5 [5.0 ; 5.9]	6.8 [5.0 ; 9.2]	5.7 [5.2 ; 7.2]	6.6 [5.2 ; 10.5]	6.6 [5.2 ; 7.5]

Item / Month	HD Modality	7	8	9	10	11	12
Number of subjects	**HHD**	57	56	53	53	55	57
	**ICHD**	54	51	49	45	48	46
S-Phosphate (mmol/L)	**HHD**	1.6 [1.2 ; 1.8]	1.6 [1.3 ; 1.9]	1.5 [1.3 ; 1.8]	1.6 [1.3 ; 2.0]	1.6 [1.4 ; 1.8]	1.6 [1.4 ; 1.9]
	**ICHD**	1.6 [1.3 ; 2.1]	1.6 [1.3 ; 2.0]	1.5 [1.2 ; 1.9]	1.6 [1.2 ; 2.1]	1.5 [1.2 ; 1.9]	1.6 [1.1 ; 1.8]
S-PTH (pg/mL)	**HHD**	443 [194 ; 612]	314 [136 ; 555]	322 [228 ; 609]	386 [189 ; 639]	346 [198 ; 520]	434 [217 ; 740]
	**ICHD**	490 [248 ; 673]	268 [183 ; 493]	261 [177 ; 527]	377 [262 ; 709]	387 [267 ; 575]	353 [207 ; 581]
S-Calcium (mmol/L)	**HHD**	2.2 [2.1 ; 2.3]	2.2 [2.1 ; 2.4]	2.3 [2.1 ; 2.4]	2.2 [2.1 ; 2.4]	2.3 [2.1 ; 2.4]	2.3 [2.1 ; 2.4]
	**ICHD**	2.2 [2.1 ; 2.3]	2.2 [2.1 ; 2.4]	2.3 [2.1 ; 2.3]	2.2 [2.1 ; 2.3]	2.2 [2.1 ; 2.3]	2.2 [2.2 ; 2.3]
S-Alkakline Phosphatase (IU/L)	**HHD**	87.5 [72.0 ; 127.0]	70.0 [59.5 ; 93.5]	82.5 [70.0 ; 120.0]	105.0 [65.0 ; 142.0]	81.0 [64.0 ; 102.0]	93.0 [69.0 ; 124.0]
	**ICHD**	84.5 [59.0 ; 146.0]	91.5 [61.0 ; 135.0]	93.0 [61.0 ; 126.0]	90.5 [60.0 ; 134.5]	83.0 [60.0 ; 124.0]	87.0 [67.0 ; 120.0]
Na (mmol/L)	**HHD**	139 [136 ; 140]	139 [138 ; 141]	139 [137 ; 140]	139 [136 ; 140]	138 [136 ; 140]	139 [136 ; 140]
	**ICHD**	138 [136 ; 140]	138 [136 ; 140]	138 [136 ; 140]	139 [137 ; 140]	139 [137 ; 140]	139 [137 ; 140]
HCO3 (mmol/L)	**HHD**	23 [21 ; 24]	22 [21 ; 24]	22 [21 ; 25]	22 [20 ; 25]	22 [21 ; 25]	22 [20 ; 24]
	**ICHD**	23 [21 ; 24]	23 [21 ; 25]	23 [22 ; 25]	23 [21 ; 24]	22 [21 ; 24]	23 [21 ; 25]
Number of subjects (Hemoglobin)	**HHD**	58	58	53	54	56	59
	**ICHD**	55	52	50	46	48	45

Item / Month	HD Modality	7	8	9	10	11	12
Hemoglobin (g/dL)	**HHD**	10.8 [9.5 ; 11.8]	10.8 [9.7 ; 11.6]	10.5 [9.8 ; 11.6]	10.5 [9.5 ; 11.5]	10.9 [9.9 ; 11.5]	10.9 [9.8 ; 12.2]
	**ICHD**	11.0 [10.1 ; 12.2]	11.0 [10.3 ; 12.3]	11.1 [10.0 ; 12.0]	11.2 [10.1 ; 11.8]	11.2 [10.3 ; 11.7]	11.1 [10.4 ; 12.2]
Number of subjects (S-Ferritin)	**HHD**	47	51	45	43	46	46
	**ICHD**	42	38	41	36	38	38
S-Ferritin (µg/L)	**HHD**	114 [39 ; 228]	96 [44 ; 227]	111 [46 ; 231]	144 [51 ; 277]	89 [49 ; 228]	80 [34 ; 215]
	**ICHD**	368 [294 ; 578]	345 [249 ; 490]	404 [295 ; 500]	373 [199 ; 481]	348 [193 ; 495]	397 [247 ; 551]
Transferrin Saturation (%)	**HHD**	19 [13 ; 27]	20 [13 ; 27]	18 [13 ; 26]	21 [12 ; 26]	20 [13 ; 26]	19 [13 ; 26]
	**ICHD**	28 [21 ; 37]	25 [18 ; 31]	25 [19 ; 35]	23 [18 ; 32]	25 [21 ; 33]	25 [17 ; 32]
Albumin (g/L)	**HHD**	41 [37 ; 43]	40 [36 ; 44]	39 [37 ; 43]	39 [36 ; 42]	41 [37 ; 44]	40 [36 ; 44]
	**ICHD**	40 [36 ; 41]	40 [37 ; 43]	40 [37 ; 41]	39 [36 ; 42]	40 [36 ; 42]	39 [37 ; 42]
Predialysis S-B2M (mg/L)	**HHD**	16.8 [14.3 ; 20.8]	20.5 [10.7 ; 25.4]	17.6 [11.4 ; 24.5]	17.4 [14.0 ; 21.8]	15.9 [12.1 ; 25.5]	17.7 [13.6 ; 27.3]
	**ICHD**	26.1 [16.7 ; 31.6]	24.5 [21.7 ; 26.1]	22.5 [17.2 ; 26.8]	24.6 [23.5 ; 30.0]	26.0 [21.8 ; 28.4]	24.5 [20.7 ; 26.2]
Hb1Ac (%)	**HHD**	4.9 [4.7 ; 7.3]	5.4 [5.0 ; 5.4]	5.5 [5.1 ; 8.0]	6.8 [5.4 ; 7.9]	5.0 [4.7 ; 6.3]	6.7 [5.1 ; 6.9]
	**ICHD**	6.5 [5.3 ; 8.8]	5.8 [4.9 ; 7.0]	7.0 [5.6 ; 7.3]	4.9 [4.6 ; 7.2]	5.4 [5.0 ; 7.0]	7.1 [6.2 ; 8.5]

Number of subjects within the table are added for parameters documented in fewer patients than indicated first row. Median [first quartile; third quartile].
FAS, full analysis set; HHD, home short-daily hemodialysis; ICHD, in-center hemodialysis.

Supplemental Table 4. Medications (FAS)

	Inclusion		3 Months		6 Months		9 Months		12 Months	
**Medications**	**HHD**	**ICHD**	**HHD**	**ICHD**	**HHD**	**ICHD**	**HHD**	**ICHD**	**HHD**	**ICHD**
Iron	39 (48%)	43 (61%)	44 (56%)	39 (61%)	46 (62%)	30 (51%)	48 (69%)	35 (61%)	44 (69%)	24 (48%)
Erythropoietin	9 (11%)	8 (11%)	9 (12%)	9 (14%)	9 (12%)	8 (14%)	9 (13%)	6 (11%)	8 (13%)	5 (10%)
Antihypertensives	55 (67%)	47 (67%)	56 (72%)	47 (73%)	50 (68%)	46 (78%)	50 (71%)	45 (79%)	47 (73%)	40 (80%)
Phosphate binders	29 (35%)	33 (47%)	30 (39%)	31 (48%)	27 (37%)	32 (54%)	25 (36%)	33 (58%)	25 (39%)	31 (62%)

n (% column) patients. FAS, full analysis set; HHD, home short-daily hemodialysis; ICHD, in-center hemodialysis.
